# Anticancer activity of quantum size carbon dots: opportunities and challenges

**DOI:** 10.1186/s11671-024-04069-7

**Published:** 2024-08-05

**Authors:** Tanima Bhattacharya, Subham Preetam, Sohini Mukherjee, Sanjukta Kar, Debanjan Singha Roy, Harshita Singh, Arak Ghose, Tanmoy Das, Gautam Mohapatra

**Affiliations:** 1 Faculty of Applied Science, Lincoln University College, 47301 Petaling Jaya, Selangor Darul Ehsan Malaysia; 2 Department of Robotics and Mechatronics Engineering, Daegu Gyeongbuk Institute of Science and Technology (DGIST), Daegu, 42988 Republic of Korea; 3 Department of Environmental Science, University of Calcutta, 35 Ballygunge Circular Road, Kolkata, West Bengal 700019 India; 4 Dietetics and Applied Nutrition, Amity University Kolkata, Kadampukur, India; 5 KIIT School of Biotechnology, KIIT University, Bhubaneswar, India; 6 Faculty of Engineering, Lincoln University College, 47301 Petaling Jaya, Selangor Darul Ehsan Malaysia; 7 Centre for Biotechnology, Siksha O Anusandhan (Deemed to be University), Bhubaneswar, Odisha India

**Keywords:** Anticancer, Carbon dot, Quantum size, Therapeutic, Biocompatibility

## Abstract

Research into the anticancer activity of quantum-sized carbon dots (CDs) has emerged as a promising avenue in cancer research. This CDs delves into the opportunities and challenges associated with harnessing the potential of these nanostructures for combating cancer. Quantum-sized carbon dots, owing to their unique physicochemical properties, exhibit distinct advantages as potential therapeutic agents. Opportunities lie in their tunable size, surface functionalization capabilities, and biocompatibility, enabling targeted drug delivery and imaging in cancer cells. However, we include challenges, a comprehensive understanding of the underlying mechanisms, potential toxicity concerns, and the optimization of synthesis methods for enhanced therapeutic efficacy. A succinct summary of the state of the research in this area is given in this review, emphasizing the exciting possibilities and ongoing challenges in utilizing quantum-sized carbon dots as a novel strategy for cancer treatment.

## Introduction

Cancer poses a significant challenge in modern medicine, prompting the need for innovative approaches in both diagnosis and treatment [1]. The field of cancer research has been transformed by nanotechnology, introducing advanced tools for precise diagnosis, imaging, and treatment of malignancies. Among various nanomaterials explored for potential use in cancer therapy, quantum-sized carbon dots have recently gained considerable attention [2–4].

These tiny carbon structures, typically ranging from 1 to 10 nm, belong to a unique class of nanomaterials with versatile properties suitable for biomedical applications [1]. Their appeal lies in the ability to control their size and surface chemistry precisely, allowing customization of key properties like solubility, stability, and bioavailability. Additionally, the surface functionalization of carbon dots enables tailored interactions with biological entities, facilitating targeted drug delivery and improved cellular uptake [5]. These inherent features make quantum-sized carbon dots a promising platform for developing advanced cancer therapeutics [6–9].

CDs derived from various structures can manifest as amorphous as well as graphitic. These CDs' sizes are modifiable through the utilization of diverse nanocomposites. Extensive research has been conducted on CDs, exploring their diverse applications. TEM and XRD are indispensable tools in the characterization toolkit for carbon dots, providing critical insights into their structural properties and helping researchers tailor their synthesis methods for specific applications [10–12]. In-depth analysis involves the application of IR spectroscopy and elementary profiling, aiding in the identification of functional moiety present on the surface that define CDs' binding characteristics. These properties, along with chemical mechanisms, offer insights for potential expansion. Additionally, CDs exhibit UV–Vis spectrum absorption, a crucial aspect for evaluating the excitation band in fluorescence spectrum studies. This spectral analysis enables the observation of alterations in CDs' surface properties, arising from interactions with other materials or derivatization, thereby influencing fluorescence intensity. Notably, the fluorescence emission of CDs serves as a valuable tool for label-free probing of targets under a microscope.

The documented mean size of CDs, measuring smaller than 10 nm, has enabled their application in all biological contexts. Passivation on the exterior of CDs are found to enhance fluorescence. The introduction of hydroxyl groups to the carbon dots resulted in CDs with a measured diameter of 3.1 ± 0.5 nm, characterized by uniform dispersion and a spherical shape as confirmed by TEM analysis. These findings highlight the impact of surface functionalization on the structural and potentially functional properties of carbon dots, making them suitable for diverse applications in nanotechnology and beyond. The quantum yield (QY) demonstrated an increase, reaching approximately 5.5%.

**Fig. 1:**
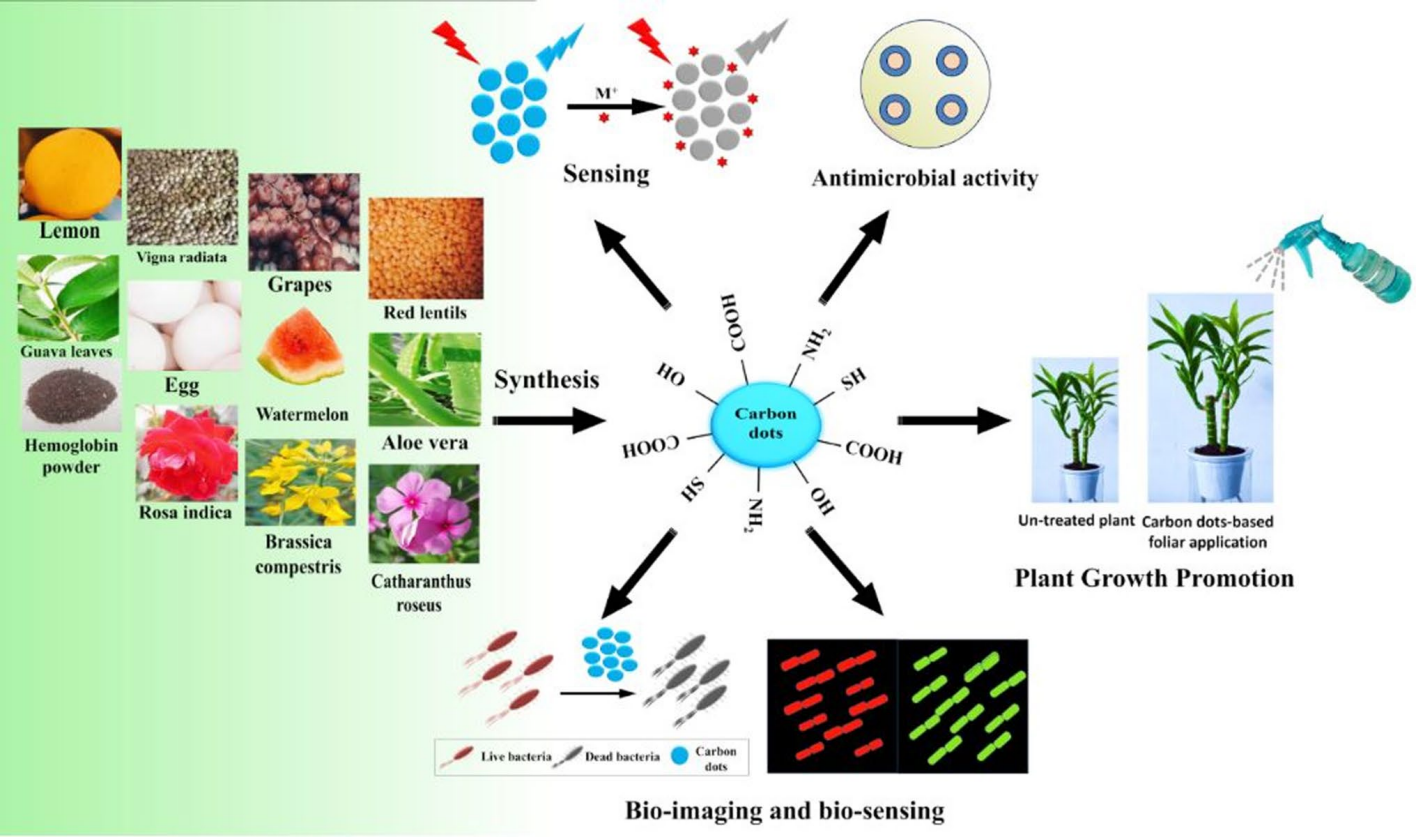
Various natural resources used for the production of CDs and their applications. Reproduced with the CC BY license from [16]

Improved fluorescence emission was observed, coupled with enhanced photostability of the particles [13]. Synthesizing carbon dots from bananas resulted in nanoparticles with an average size of 3 nm. This approach utilizes natural sources to produce nanomaterials with potential applications in various fields, leveraging the inherent properties of the starting material for tailored functionalities [14, 15]. These spherical carbon dots primarily consist of carbon and oxygen, with a minor potassium content. Their interlayer spacing, involving oxygen-containing groups linked to sp3 carbon and hydroxyl-connected carbon groups, measures 0.42 nm, which exceeds the graphitic interlayer spacing of 0.33 nm, indicative of a diamond-like structure. The fifth group of carbon dots, derived from multiwalled carbon nanotubes via electrochemical oxidation, exhibited a graphitic structure with a lattice spacing of 3.3 Å, akin to the (002) facet of graphite. This structural similarity highlights their potential for various technological applications based on their unique properties. CDs encompass a diverse range of nanomaterials with unique properties and applications. Derived from sources such as candle soot and multiwalled carbon nanotubes via electrochemical oxidation, CDs exhibit varying compositions and structures. CDs from candle soot display high oxygen content and feature functional groups like hydroxyl, carbonyl, and carboxyl, with sizes ranging from 2 to 7 nm. They are promising for drug delivery due to their small size and large surface area. In contrast, CDs formed with BiVO_4_ nanocomposites show sizes ranging from 350 to 500 nm and combine fluorescence with semiconductor characteristics for applications in photocatalysis and solar energy conversion. Adopting a standardized nomenclature is crucial for clarity in research and development efforts concerning CDs, ensuring accurate classification and application-specific synthesis (refer to Fig. 1).

Carbon nanodots (CNDs) comprise various types: carbon nanoparticles (CNPs), which lack a defined crystal lattice and do not exhibit the quantum confinement effect (QCE); carbon quantum dots (CQDs), with well-defined structures and multiple graphene layers, showing QCE due to their nanoscale size; and graphene quantum dots (GQDs), featuring diverse shapes and π conjugation within graphene sheets, contributing to unique electronic properties including edge effects. These classifications are crucial for understanding how CNDs' structural and chemical diversity influences their optical and electronic characteristics, essential for applications spanning biomedical imaging, catalysis, and optoelectronics. Therefore, the fluorescence mechanisms differ among various types of CDs. In carbon nanoparticles (CNPs), photoluminescence (PL) primarily arises from molecular, subdomain, and surface states [13, 17–21].

Research into quantum-sized carbon dots as agents with inherent anticancer properties has gained significant momentum. Studies have shown that these nanomaterials can induce apoptosis, suppress proliferation, and selectively target various types of cancer cells [22]. These effects are typically driven by intricate interactions at cellular and molecular levels, offering valuable insights into potential pathways for cancer intervention. This section explores the expanding evidence supporting the innate anticancer activity of quantum-sized carbon dots.

In addition to their intrinsic anticancer properties, CQDs excel as transporters for drug delivery. Incorporating therapeutic agents onto or into these nanomaterials enables controlled release and targeted delivery at the tumor site, minimizing off-target effects and improving treatment efficacy [23]. Additionally, the exclusive photophysical characteristics of carbon dots position them as optimal candidates aimed at bioimaging applications [24]. Their fluorescence and photoacoustic capabilities enable precise visualization of tumors, contributing to both diagnosis and treatment monitoring.

As interest in quantum-sized carbon dots continues to grow, this review seeks to offer a inclusive synopsis of the opportunities and challenges associated with their application in cancer therapy. Through a careful examination of recent advancements, key findings, and critical gaps in knowledge, we seek to offer insights that will guide future research endeavors in fully realizing the potential of these remarkable nanomaterials.

## Carbon dots: a brief overview

Singular-layer carbon nanotube purification resulted in the discovery of carbon dots, a distinct category of carbon nanomaterial [25]. Nanostructures are materials that exhibit dimensions at the nanoscale, typically ranging from 1 to 100 nm. Carbon dots (CDs) are distinct, discrete, sub-micron-sized molecules that are entirely spherical and zero-dimensional with semi-conductor like properties. They are less than 10 nm [26] with their excellent physico-chemical properties owing to their many advantages; including their small size, good quantum yield, abundant availability, good tensile strength, UV barrier, satisfactory photostability, low toxicity, good biocompatibility, cost efficiency, simple synthesis process, high water solubility, bioactivity, and light absorption, antioxidant, antimicrobial and photoluminescence capacity; Carbon dots (CDs) find extensive use in diverse medical applications, which not only improves the economic benefits but also lessens environmental pollution.

### Definition and characteristics

CDs are zero-dimensional, quasi-spherical nano-particles less than 10 nm in size. They are considered as the ‘perfect’ carbon nanomaterial because of its exceptional high yield, low toxicity, light stability, superb biological compatibility, and least cost for synthesis [27]. Carbon nanodots (NCDs), CQDs and GQDs are the typical classifications for CDs. Modifying the surface of carbon nanoparticles with polymeric and organic compounds is necessary for CD production. To create CDs, a variety of wastes are used as carbon sources, such as orange peels, tea stems and common carbon dot sources for food applications include some environmentally favourable ones, like paper waste, plant extracts, fruit juice, residual tea waste, and foods of animal or plant origin (honey, meat, butter, seafood varieties, etc.). This decreases environmental pollution and enhances the economic benefits [28]. The three principal areas of CD study are metal ion adsorption, fluorescence detection, and wound healing [29]. Furthermore, it has been shown that CDs possess many antibacterial properties, with their antibacterial mechanisms involving oxidative stress, physical damage, and suppression in the metabolic process of bacteria [30]. Food packaging can benefit from the additional usage of carbon dots (CDs), which are made of green sources and extend the shelf life by retaining quality and freshness of food items [31–33]. Carbon dots are easily manufactured from waste or environmentally favourable products including fruits, peels, leaves, and honey (Fig. 1). The use of such active materials in food packaging not only improves the functionality of packaging but also contributes to economic benefits and sustainability by reducing environmental impact through efficient food preservation and waste reduction [34].

Unique electrochemical and optical characteristics of CDs make them widely acceptable in biomedical fields, in various sectors like biosensors, bioimaging, drug and gene delivery and therapeutic development in the field of photodynamic/photothermal therapy.

- Stability Enrichment: Because of their prolonged chemical stability in diverse solvents and the abundance of functional groups on their surfaces, CDs have been demonstrated as superior nanomaterials for chemical durability of hybrid catalysts [35]. Additionally, CDs' electrostatic stabilization can help hybrid catalysts function more steadily by demonstrating high stability in aqueous conditions.
  1. Size-dependent properties: Optical and electronic properties of CDs, including their behavior, strongly correlate with their size. Smaller-sized dots tend to exhibit higher energy emissions and broader absorption spectra.
  2. Quantum confinement effect: When carbon dots are small enough, typically below 10 nm in diameter, they experience quantum confinement effects. This effect leads to discrete energy levels, resulting in tunable photoluminescence and electronic properties.
  3. Photoluminescence: Carbon dots have a notable photoluminescent property, which means they can absorb light energy and re-emit it in the form of fluorescence [36]. The emission wavelength of CDs can be tailored by adjusting their physicochemical characteristics.
  4. Electrical Conductivity: CDs exhibit excellent electrical conductivity. This characteristic facilitates the removal of the Schottky barrier that forms at the junction of the electrolyte and catalyst in electrocatalytic systems, thereby enabling efficient energy transformation. Moreover, CDs facilitate rapid electron transfer during electrochemical reactions due to their superior electrical conductivity [37].
  5. Biocompatibility: Carbon dots are considered biocompatible and exhibit low toxicity, making them suitable for biological applications. They find applications as fluorescent labels in drug delivery vehicles, imaging, and sensors for biomedical research and diagnostics.
  6. Water dispersibility: Carbon dots possess excellent water dispersibility due to their hydrophilic surface functional groups. This characteristic makes them highly soluble in water and facilitates their utilization in aqueous environments.
  7. Excitation wavelength dependence: The absorption and emission properties of carbon dots can vary with different excitation wavelengths This is due to the existence of multiple emission centers or energy states within the dots.
  8. Environmental sensitivity: It is possible to engineer carbon dots so that they react differently to different environmental conditions, such as pH, temperature, or the presence of ions or molecules. This sensitivity can be utilized for sensing and monitoring applications.
  9. Emission properties: By adjusting physicochemical parameters during the synthesis of CDs, one can vary the excitation wavelength of the discs and produce a variety of fluorescence emissions. The emission, influenced by pH, is caused by the hydronation and dehydronation of surface functional groups; The fluorescence dependence on concentration is attributed to surface emission, while temperature dependent emission is caused by nonradiative degradation on surface of CDs [5].

Specific characteristics and properties of carbon dots are impacted by the synthesis method, surface functionalization, and intended applications. Ongoing research and advancements in carbon dot synthesis techniques continue to explore and uncover new possibilities for these nanoscale carbon materials.

### Synthesis methods

**Fig. 2:**
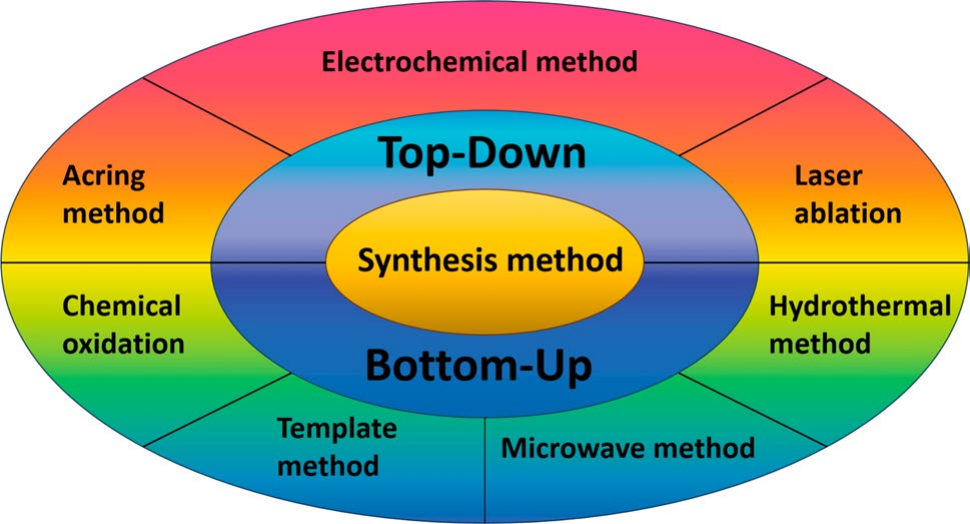
Approaches for synthesizing carbon dots. Reproduced with CC-BY License from [40]

During the manufacture of CDs, a range of functional groups can be added, including hydroxyl, carboxyl, carbonyl, amine and epoxy etc. [38]. Moreover, doping CDs with heteroatoms, using various biological, polymeric, and organic materials allows for easy surface functionalization [39, 40]. Moving forward, the properties of CDs can be controlled by altering physicochemical properties using various precursors or alternative synthetic methods. To achieve significant surface properties for solvency and their advantageous applications, CDs must be modified [40]. The synthesis of carbon dots involves several methods, each with its own variations and approaches. Here are a few commonly used techniques for synthesizing carbon dots (Fig. 2).

#### Top-down method

These days, it's common practise to use top-down methods like electrochemical processes, laser ablation, arc discharge, and ultrasonic treatment to create CDs from bulk carbon-based materials. Nevertheless, these techniques are usually used in high energy, high potential, and high acidity settings. These top-down methods are arduous due to the harsh conditions, as opposed to bottom-up processes [41].

Arc Discharge Method: Although CDs produced by arc discharge with QY, the arc discharge method remains viable for synthesizing CDs from basic carbon nanotubes. The initial CDs were generated by single-walled and multi-walled carbon nanotubes through an oxidation process utilizing the arc discharge approach [42, 43]. Moreover, Arora and Sharma said that the C-atoms that are created by breaking down large C-precursors can be reoriented using the arc discharge process to get high-energy plasma inside the reaction apparatus.

Laser Ablation Method: The process involves focusing a high-energy laser beam onto a target material, which in this case is a bulk carbonaceous material. The laser pulse is intense and short-duration, typically in the nanosecond to picosecond range. When the laser pulse strikes the surface of the carbonaceous material, it rapidly heats a small portion of the material. The intensity of the laser pulse is sufficient to vaporize or ionize the carbon atoms in that localized area. Heat levels rise as a result, and plasma begins to evaporate. After that, a crystallization procedure turns the produced vapor into CDs. Sun and the team demonstrated the creation of luminescent CDs by employing argon gas for water vapor and a carbon target through laser ablation technique [44].

Ultrasonic Method: According to [45], this technique produces tiny vacuum bubbles in the solution by applying ultrasonic waves at low and high pressures. As a result, it inhibits accumulation, leading to a stout hydrodynamic shear energy and quick liquid jet contravention. Graphite, activated carbon, and CNTs are examples of large-sized carbon-based nanomaterials that can be broken down into nanosized CDs using the energy generated by the ultrasonic process.

Electrochemical/Chemical Oxidation Method: This process is widely preferred for creating CDs due to its numerous benefits. When used, this method produces extremely unadulterated CDs with a high QY suitable for large-scale production, and it is remarkably fast, cost-effective, and consistent. It also allows for easy control over CD size [46]. The CDs are produced chemically or electrochemically using oxidation–reduction processes at room temperature and pressure. Strong oxidising agents including corrossive acids (HNO_3,_ H_2_SO_4_ etc.), and hydrogen peroxide (H_2_O_2_) are used in it. By controlling the redox processes and the electrolyte components, hydrophilic groups like -NH_2_, -COOH, -OH, etc., can be functionalized on the surface of CDs.

#### Bottom-up approach

Given their many benefits, including their straightforward and convenient technique, cost-effectiveness, precise control, easy instrumentation, and promising practical applicability—bottom-up approaches are currently in vogue.

#### Thermal method

One of the best techniques for creating CDs is thermal breakdown, which entails pyrolyzing or carbonizing the large-sized carbonaceous precursors at higher temperatures. This approach has several advantages, including production of CDs in copious quantities, affordability, shorter reaction times, increased precursor tolerance, solvent-free techniques, and simplicity of synthesis [47]. Furthermore, the thermal technique can optimize the luminous properties of CDs by regulating reaction conditions.

#### Microwave-assisted method

Microwaves encompass a broad spectrum of electromagnetic waves ranging from 1 mm to 1 m [48]. The microwave-assisted approach generates homogenous temperature for the even dispersal of CDs and is simple, inexpensive, quick, and requires shorter reaction times.

#### Hydrothermal method

This method of CD synthesis is economical, non-toxic, and ecofriendly. It entails the sealing of organic solvents as precursors and their reaction at greater pressures and temperatures in a hydrothermal reactor. The hydrothermal carbonization (HTC) process is capable of producing carbon dots (CDs) from a variety of raw materials, including proteins, chitosan, citric acid, glucose, and more. [49].

#### Template method

Two steps are involved in the synthesis of CDs utilizing the template method: (i) calcining the desired CDs in a mesoporous silicon sphere or a suitable template, and (ii) an etching step to remove supports. Nevertheless, the synthesis of CDs has not made greater use of this technology [45].

### Quantum size effects

QDs are semiconductor crystals with size from 1 to 10 nm. The quantum confinement effect (QCE) causes these crystals to exhibit optical features including fluorescence [50]. The qualities of quantum dots (QDs) are dependent on their size, and as the size declines, so does the emission wavelength (λem).

The electrons in carbon dots are confined within their tiny nanoparticle structure, which leads to quantum size effects. The electrons' energy levels and mobility are restricted by this confinement, which causes their properties to become quantized. For instance, because of their confinement, electrons in carbon dots smaller than 10 nm in size have unique and discrete energy levels. As a result, the energy band structure and electronic transitions of carbon dots differ from those of bulk carbon materials. The distinct optical or electronic characteristics of carbon dots, such as increased fluorescence, adjustable emission wavelengths, and better photochemical stability, are a result of these quantum size effects [51]. Carbon dots are interesting materials for sensing, imaging, and optoelectronics because of their quantum size effects. Moreover, carbon dots' catalytic capabilities are also influenced by their quantum size effects. Carbon dots become effective catalysts for a variety of chemical processes due to these properties, which also increase their catalytic activity. Researchers have devoted significant attention to carbon dots (CDs) due to their exceptional properties. These include an excellent electron conductivity, resistance to photodecomposition, variable excitation and emission characteristics [52].

Since their surface is covered in many functional groups, CDs are easily functionalized. They also have exceptional sensing properties, including multiplex, selective, and specific detectability. Due to their surface being adorned with numerous functional groups as amine, carboxyl, hydroxyl, etc., or polymer chains, CDs exhibit high solubility in aqueous solutions. Additionally, this facilitates their functionalization with additional nanomaterials [53].

### Surface functionalization

The goal of NP surface functionalization is to enhance and add features that will make NPs more beneficial for usage in the field of medicine. Various kinds of nanomaterials can be used in the initial stages of functionalization because of their exposed functional groups and distinctive chemical characteristics. Depending on the patient's age, condition, location, kind of tissue, and disease stage, a variety of medicines are currently accessible for cancer therapy [54, 55]. While radiation, surgery, and traditional chemotherapy drugs are still effective and frequently used to treat tumors, their adverse effects are frequently quite concerning [23, 56]. In carbon-based nanomaterials, carbon atoms are in the sp^2^ hybridized state [57]. Furthermore, carbon atoms allows for the easy attachment of functional groups like hydroxyl, carboxyl, and amine groups [58]. both halogenation and cycloaddition are chemical reactions used to modify the surface of nanoparticles. Halogenation introduces halogens which can further react with other groups like amines to form new functional structures, while cycloaddition allows for the addition of a variety of functional groups directly. These modifications can tailor the properties of nanoparticles for specific uses in different fields like medicine, electronics, and materials science [59].

Anatomical perception of the light-induced antibacterial action of CDs is being sought through the investigation of several plausible mechanisms that bear similarities to the action mechanisms of other nanoparticles. The mechanisms by which carbon dots potentially exert antibacterial effects, including adhesion to cell membranes, ROS production leading to oxidative damage, cellular penetration affecting functions, and modulation of cell signaling. One proposed theory suggests that in low-light conditions, CDs generate ROS like singlet oxygen, superoxide, and hydroxyl radicals to effectively combat bacteria [60].

In the context of CDs, the surface passivation or functionalization of their core–shell architecture is crucial, as it profoundly influences key material attributes, particularly optical qualities and surface charge status. One of the most crucial characteristics influencing how nanomaterials interact with biological entities is surface functionalization. This, in turn, influences the nanomaterials' cytotoxicity, intracellular trafficking, and cellular uptake pathways [61]. The surface charges on the nanoparticles may have a significant role in influencing the interactions between biological entities and nanomaterials, which rely on the electrostatic interactions. The physical, optical, and photo-induced features of CDs, including their thickness, fluorescence quantum yield, and photo-induced antibacterial activities, are also significantly influenced by the surface passivation layer that is created by various molecules. For this reason, CDs are structurally adaptable and can have their surface functions altered to enhance their ability to interact with bacterial cells and their antibacterial properties.

The surface of nanoparticles can be modified through two main methods, such as non-covalent and covalent association. The non-covalent method relies on various fragile bonding, including ionic, electrostatic, hydrophobic and van der Waals contacts, as well as adsorption and hydrogen bonding. This approach is particularly useful for modifying silica and metal based nanoparticles [62, 63]. There is an advantage of non-covalent bonds, that they are straightforward; they don't alter the molecules' structures or their interactions with biological targets. Nonetheless, a variety of variables, such as ionic strength and pH, can affect non-covalent modifications [64]. Nanoparticles (NPs) are characterised by their size, shape, charge, composition, and surface chemical groups, which all have an impact on their toxicity and absorption efficiency. Two types of surface modification that can be easily changed are chemical groups and surface charge.

## Anticancer potential of carbon dots

**Fig. 3:**
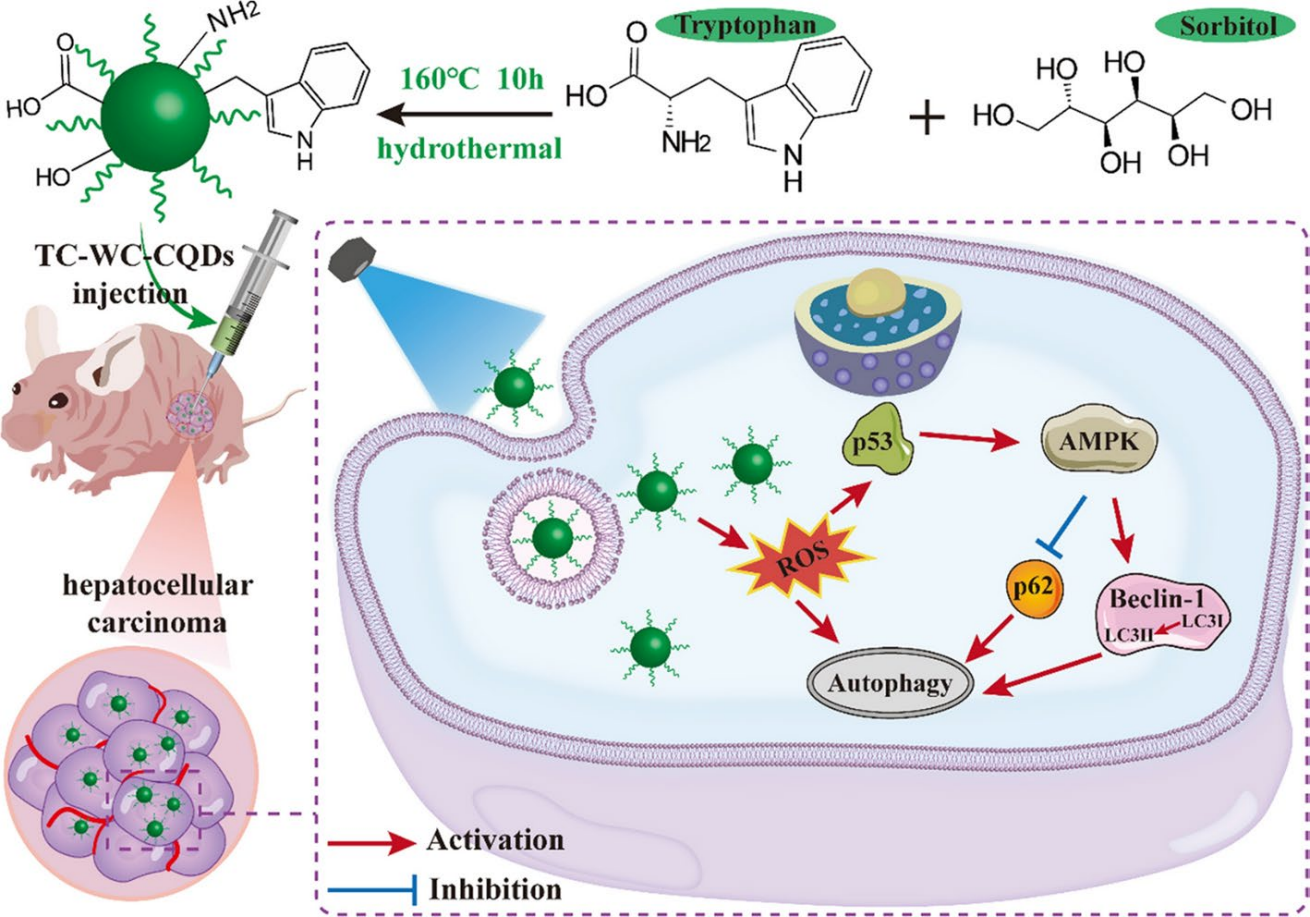
Diagrammatic depiction of the utilization of CQDs in the diagnosis and treatment of hepatocellular carcinoma Click or tap here to enter text. Reproduced with Reproduced with CC-BY License from BMC (2022) [69]

Cancer stands out as a highly impactful illness in human lives. The World Health Organization has ranked it as the leading (primary or secondary) cause of death before 70 years of age in many developed nations [6, 65]. It is estimated that by the year 2040, there will be 27.5 million newly detected cancer cases and an anticipated 16.2 million deaths [66]. Cancer arises from uncontrolled cell growth, forming abnormal masses or tumors. Despite medical advancements, elevated mutation rate and the process of metastasis challenge early diagnosis and treatment [67]. Common cancer treatments, such as radiotherapy and chemotherapy, exhibit serious side effects like cell death, hair loss, and blood-related issues [68]. Consequently, there is a pressing need for a more precise and less invasive approach to cancer detection and treatment [65]. Carbon quantum dot (CQD)-based strategies has been crucial in advancing cancer treatment and diagnosis, which includes transportation of drugs, bio-imaging, nanomedicine shown diverse applications over the years. According to the study of Wang et al., the research introduced a novel approach for targeting and treating hepatocellular carcinoma (HCC) using bio-friendly carbon quantum dots (CQDs), specifically trichrome-tryptophan-sorbitol based CQDs. By developing a single-pot hydrothermal method, these CQDs were produced from a naturally sourced tryptophan. Notably, these carbon quantum dots exhibit a formation of fluorescence of bright green colour, which is significantly more pronounced in HCC cells than in normal hepatocytes, highlighting their selective targeting ability. Furthermore, the green fluorescence releasing CQDs produce significant amounts of reactive oxygen species, which induce autophagy in HCC cells [69]. Further studies demonstrate that these CQDs effectively inhibit tumor growth with minimal systemic toxicity, indicating their potential as a safe and efficient treatment for HCC (Fig. 3) [69].

CQDs play a pivotal role in advancing anti-cancer nanodrugs, employing a versatile approach to combat cancer through multiple avenues. These minute carbon-based nanoparticles contribute to a theranostic strategy, a groundbreaking method that tailors treatment to individual patient characteristics, reshaping the landscape of disease management, particularly in cancer. This innovation leads to more precise and efficient interventions in medication delivery, diagnosis, and therapy [70]. Leveraging their fluorescence and biocompatibility, CQDs serve as valuable imaging agents in early cancer detection. Furthermore, with surface-attached ligands, CQDs serve as targeted carriers for medications, delivering chemotherapeutic drugs specifically to cancer cells. Their theranostic properties enable seamless integration of real-time imaging with therapy, facilitating dynamic treatment monitoring. This article segment delves into the diverse applications of CDs in the development of anti-cancer nanodrugs.

### Mechanisms of action

Fluorescence imaging of cells is a used tool that is extensively used in biomedicine. CQDs, with their bright and customizable fluorescence, are suitable for bioimaging [71]. Special ligands like folic acid, hyaluronic acid, and transferrin have been attached to CQDs for targeted staining in cell imaging, particularly beneficial in cancer cells overexpressing the folate receptor [72]. Using folic acid as a precursor, luminous CDs exhibiting a substantial quantum yield of 95% has been successfully synthesized for effective cancer cell targeting and imaging [73].

Recent interest in developing nano-scale drug delivery systems designed for anti-cancer therapy arises from the limitations of current inexact, inadequately soluble drugs with numerous adverse reactions. CDs, owing to their excellent water solubility and biodegradability, serve efficiently as anti-tumor nano-scale drug carriers. Attachment to molecules occurs through covalent or non-covalent bonds, offering controlled release options [2]. For instance, Zeng et al. synthesized CD-Doxorubicin conjugates with pH-sensitive release, utilizing hydrogen bonding between carboxyl and amine groups [74]. CDs being doped with phosphorous and nitrogen,have been introduced with improved loading capability of drug, employing a central hollow space and various functional moieties [75]. In a study, Jha et al. created DNA-derived CDs (Biodots) for non-small lung cancer treatment. They designed liposomes with ETP and cetuximab-conjugated DNA CDs for targeted tumor cell therapy [76].

Nominal harms of healthy tissues have been observed in case of photoinduced therapy, which offers a non-invasive approach with great specificity [77]. The treatment comprises photothermal therapy (PTT) using Near-Infrared light for controlled tissue temperature elevation and photodynamic therapy (PDT) employing photosensitizers to generate reactive oxygen species, inducing oxidative DNA damage in cancer cells. Notably, cancer cells exhibit increased H2O2 levels due to mitochondrial superoxide dismutase overexpression, making H2O2 a pro-drug for selective cancer cell damage through oxidative DNA damage when activated appropriately [78].

### In vitro studies

CQDs demonstrate excellent performance in bioimaging, good biocompatibility, boasting low toxicity, bright fluorescence, and enhanced penetration. Significantly, negligible cytotoxicity found to be observed at a concentration of 0.4 mg/ml, making them safe for fluorescence imaging [79]. Successful in vitro studies have imaged various cancer cell types, including MCF-7, HeLa, and c6 Glioma, using CQDs [80]. Innovative approaches, such as the development of CQDs from DNA molecules by Guo et al. and acid-catalyzed synthesis by Wang et al., showcase bright fluorescence and reduced reaction times [81]. According to Wang et al., functionalization of CQDs with MUC-1 aptamer for precise targeting of MCF-7 human breast cancer cells were found to be observed through binding with the MUC-1 cancer protein.

CD-Asp, an intelligent nanomedicine derived from D-glucose and L-aspartic acid, possesses the capability to traverse the barrier of blood–brain and can function as a probe for fluorescent imaging, designed for particular c6 glioma cells [82]. In a study, Jung and colleagues presented citric acid and β-analine based zwitterionic CQDs, showcasing multicolored fluorescence and effective penetration into Hela cell cytoplasm through endocytosis, facilitated by the functional groups with a positive charge [83].

Protein-based CQDs (PND) from lysozyme, which are synthesized incorporating melatonin for its anti-tumor and antioxidant properties, result in melatonin-loaded PNDs (MPND) which demonstrate effective cellular uptake in cells associated with breast cancer [84]. Ginsenosides, the primary compound in Panax ginseng, are extensively utilized in Asian and Western herbal medicine. Renowned for their medicinal properties, ginsenosides exhibit anti-inflammatory, anti-cancer, and anti-oxidative effects. Innovatively ginsenoside versatile carbon dots with multiple functionalities have been synthesized that are multifunctional and demonstrate precise suppression of cancer cells by raising levels of Reactive Oxygen Species (ROS) and triggering a ROS-mediated apoptotic pathway [85]. Camellia Japonica, a plant rich in bioactive compounds [86], and based on this study carbon quantum dots doped with sulfur (S-CDs) were synthesized for effective photothermal therapy [87].

### In vivo studies

Utilizing nano-based materials for diagnostic purposes in vivo and targeted therapy requires meeting stringent criteria beyond cell labeling. Furthermore, with minimal toxicity and effective cellular uptake, key parameters include hydrodynamic diameter, hemocompatibility, rapid extraction from the body, and small minimal nonspecific protein adsorption, ensuring efficient transportation to the specific target tissue, typically cancerous [88].

Zebrafish embryos have gained attention for assessing CD toxicity, given their genomic similarity to humans, brief generation time and optical clarity enabling real-time CD detection for bioimaging [89, 90]. Curcumin-derived CDs were found to be non-toxic in the zebrafish model. The CDs also showed multicolor fluorescence, high photostability along with biocompatibility and were useful in the bioimaging of zebrafish embryos [91].

Researchers are increasingly focusing on tumor visualization to enhance comprehension of metastatic mechanisms, accurate diagnosis, and treatment. Currently, near-infrared (NIR) imaging is widely utilized for high-resolution early-stage tumor diagnosis [92]. Despite relatively weak emission found to be observed in the near-infrared region, CDs show significant capability for conducting in vivo fluorescence tracking studies. Yang et al. introduced CDs and ZnS-doped carbon dots (CDs) employed as contrast agents in live mice through laser-ablated nanomaterials. Modified with PEG_1500N_, these agents utilized for optical imaging in vivo. Injection into mice showed slow diffusion, and fluorescence faded 24 h **post**-injection [93].

**Fig. 4:**
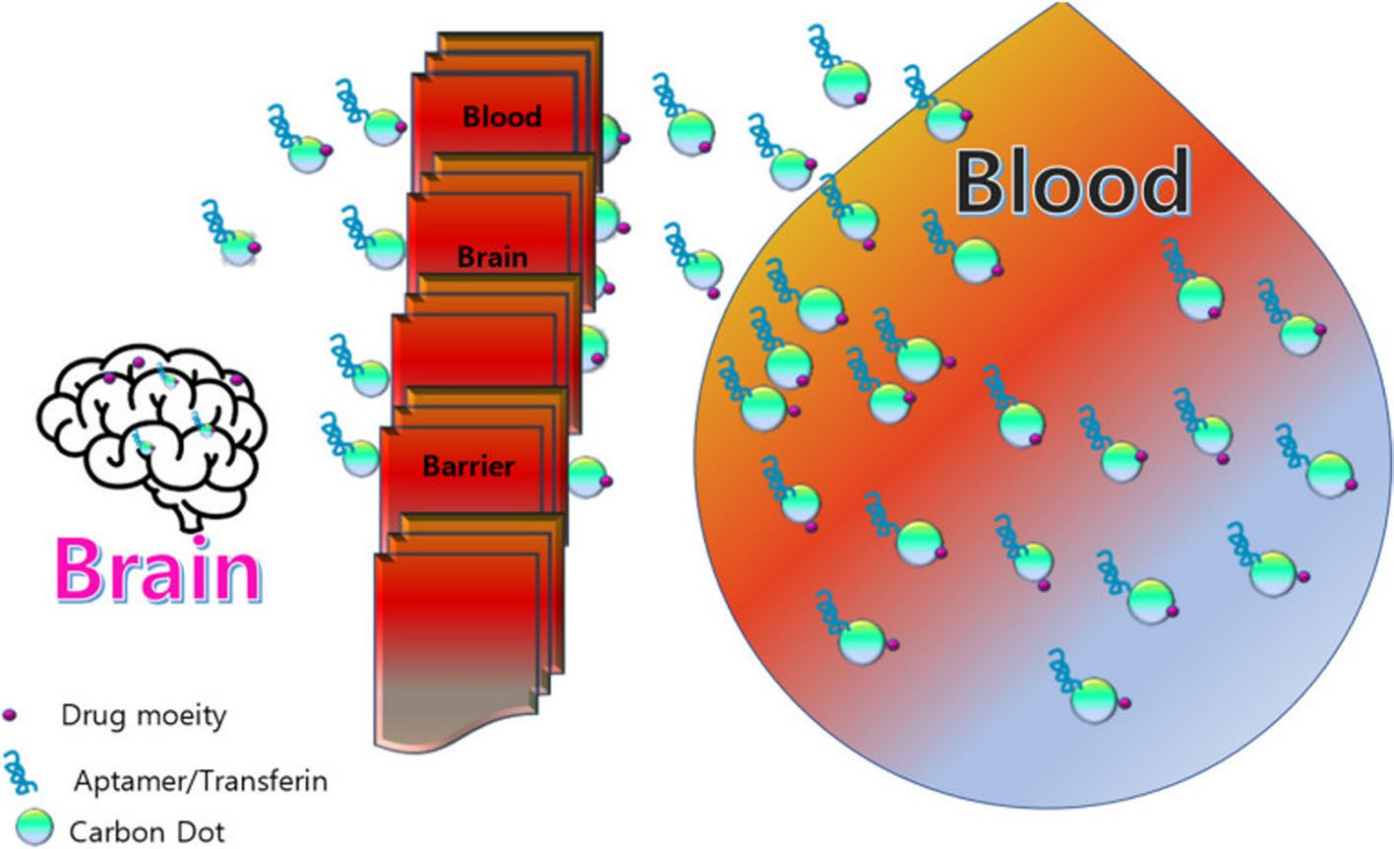
Diagram of CDs as carrier of nano drug for brain, crossing the blood–brain barrier (BBB) for enhanced therapeutic effects and improved survivability. Reproduced with CC BY license from [94]

Effective brain tumor treatment faces a significant challenge due to therapies struggling to reach therapeutic levels across the blood–brain barrier (BBB) [94]. The BBB's unique microvasculature acts as a tight barrier, regulating molecule entry into the central nervous system. While tight junctions permit the passage of specific substances, maximum percentage of both small and large molecules are hindered, severely limiting available therapeutic agents for brain tumor treatment [95]**.** CDs based therapeutic strategies exhibit enhanced capability in crossing the blood–brain barrier (BBB) to transport and deliver neurological drugs into the central nervous system (CNS) in Fig. 4 [94]. Li et al. showed in vivo that transferrin-functionalized CDs can traverse the BBB, suggesting their potential for treating neurological diseases [96]. Additionally, Seven et al. reported that fluorescein-conjugated C-dots, derived from glucose, cross the BBB in zebrafish and rats without requiring an extra targeting ligand, highlighting their efficacy as a CNS drug transportation system [97].

Crucial in the context of drug delivery, modifying carbon dots (CDs) through receptor-based methods enhances cellular absorption in vivo during cancer therapy. Research shows enhanced cellular uptake of carbon dots (CDs), particularly when conjugated with hyaluronic acid (HA) as a targeted ligand that binds to CD44, which is upregulated on numerous cancer cell [98].Modified carbon dots, synthesized through hydrothermal method by citric acid and branched poly (ethylene amine), show accumulation in tumor tissue when administered intravenously. Loading DOX onto HA-CDs exhibited antitumor efficacy in two separate tumor models, presenting a hopeful prospect for the advancement of targeted cancer therapy [98]. Kim et al. utilized Japanese camellia in the hydrothermal preparation of carbon dots,doped with sulfur (S-CDs) with robust near-infrared absorption capabilities. They observed that lower doses of S-CDs demonstrated superior photothermal therapy [87].

### Advantages of quantum-sized carbon dots for targeted delivery of drugs in *cancer* cells

The context of cancer treatment, CDs are demonstrating significant potential as versatile tools. They serve multiple functions including drug delivery, therapeutic techniques, and diagnostics. CDs excel in photodynamic therapy (PDT) due to their ability to generate ROS upon exposure to specific photo wavelengths, inducing cancer cell apoptosis [99]. Additionally, CDs act as efficient drug carriers, enhancing drug solubility and enabling targeted delivery, thereby minimizing systemic side effects. Furthermore, CDs hold promise in enhancing the efficacy of radiation treatment, angiogenesis inhibition, and metastasis prevention [100]. CDs also function as imaging agents, aiding in cancer visualization and detection. Their versatility extends to gene therapy and biosensing, offering chances for targeted modulation of gene and fast disease detection [94, 99, 100].

The ability of CDs to facilitate intracellular drug release represents a significant advantage in delivery of drug. This capability is crucial for ensuring the curative effectiveness of the administered medicines. Drugs must traverse various biological barriers to reach their targeted sites [101]. CDs play a pivotal role in this process by encapsulating medications and aiding their efficient delivery to target cells, thereby enhancing the overall efficacy of drug delivery systems. In this context, targeted drug delivery emerges as a crucial strategy. While traditional chemotherapy effectively eliminates cancer cells, it also harms healthy tissues, leading to significant side effects and systemic toxicity [100, 102]. Developing drug carriers capable of delivering anti-cancer medications specifically to tumor sites offers a solution to these limitations. This approach aims to maximize therapeutic efficacy while minimizing damage to healthy tissues, improving the overall safety and effectiveness of cancer treatment [103]. By attaching specific substances or antibodies to the cancer cell surfaces, CDs can be quickly targeted toward these cells. These substances facilitate efficient and precise drug delivery by identifying and attaching to receptors that are augmented on the cancer cell surfaces. This targeted approach enhances the effectiveness and specificity of drug administration while minimizing damage to healthy tissues [99]. A crucial element of targeted delivery of drug is controlled and precise release of anti-cancer medicines directly at tumor locations [94]. This capability ensures that drugs are released at the right place and time, maximizing their therapeutic impact while minimizing systemic side effects.

In cancer treatment, addressing multiple facets of the disease—like suppressing tumor growth, enhancing immune response, and inhibiting angiogenesis—often involves combining different medications. CDs are well-suited for combination therapy because they can simultaneously transport multiple drug payload [94, 104]. Combining medications with diverse mechanisms of action can significantly improve treatment efficacy. For example, a CD-based combination therapy might include a drug to enhance the immune response of the body against the tumor, alongside an agent act as chemotherapeutic drug that targets rapidly dividing cancer cells. This integrated approach offers a comprehensive strategy for cancer treatment, maximizing therapeutic benefits while potentially reducing the risk of resistance development [105]. Yifang et al. developed CQDs loaded with DOX using electrostatic interactions, demonstrating pH-sensitive drug release superior to free DOX. This formulation showed increased toxicity against adenoid cystic carcinoma cell lines, referring enhanced effectiveness in treating cancer. Amirhossein et al. utilized CQDs conjugated with polyamidoamine (PAMAM) for delivering genes in treating breast cancer [106]. According to Yang et al., DOX associated with CQDs modified as a DOX-CDs, showed enhanced efficacy in tumor suppression compared to free DOX in A549 and MCF-7 cell lines [100, 107]. Xudong et al. developed positively charged CDs using polyethyleneimine and folic acid, facilitating bioimaging of positive folate receptors and targeted delivery of therapeutic plasmids to specific cells. Tao et al. constructed a dual-responsive complex from CQDs, RGD peptide, monomethoxy polyethylene glycol, and a cisplatin prodrug, demonstrating therapeutic efficacy via integrin v3 receptor-mediated uptake of nanocarriers [108].

In recent years, significant advancements have been witnessed in the utilization of CDs for photodynamic therapy (PDT). In PDT, CDs cause cancer cell death from ambient oxygen when exposed to specific light wavelengths. This process operates through a multistate sensitization mechanism. For instance, NIR-emitting CDs derived from pheophytin powders show notable capabilities in producing singlet oxygen, suggesting promising applications in cancer treatment [10, 94].

In contrast, PTT employs photothermal agents that convert photon energy into heat, effectively eradicating cancer cells. NIR-emitting CDs designed for PTT demonstrate high efficiency in converting light into heat, exhibit broad absorption spectra, and emit strong fluorescence. These attributes significantly enhance their potential as effective tools in cancer therapy [100].

While PDT and PTT offer benefits in cancer treatment, practical challenges remain. For instance, PDT's efficacy is hindered by the hypoxic conditions within tumors, while PTT often causes collateral damage to surrounding healthy tissues. Addressing these issues requires specialized CD-based therapies.

Yet, these treatments often lack sufficient tumor-targeting efficacy for clinical application. Recently, CD receptor-mediated treatment methods have emerged to tackle these challenges. By surface-functionalizing CDs with specific ligands, they can precisely target receptors overexpressed on cancer cell membranes, ensuring precise drug delivery. This receptor-based modification is crucial for enhancing the cellular uptake of CDs during the treatmen of cancer [94, 100].

For instance, the ligand hyaluronic acid (HA) targets CD44 receptors, which are commonly overexpressed on various types of tumor cells. This targeted approach holds promise in improving the efficacy and specificity of cancer treatment using CDs.

CDs play dual roles: they act as carriers for targeted drug delivery and function as photosensitizers in PDT [94, 100]. Shahshahanipour et al. investigated CDs clathrates loaded with methotrexate and functionalized with folate receptors for targeted delivery of drug, alongside PDT for treating tumors.Using an innovative method, CDs synthesized from Lawsonia inermis (henna) by Shahshahanipour et al. exhibited exceptional stability and fluorescence properties without surface functionalizatio [109]. These henna-derived carbon quantum dots served as a biologically compatible fluorescent probe to detect methotrexate in human serum via the FRET mechanism. Meanwhile, Qianqian et al. formulated CQDs for delivering doxorubicin (DOX) with integrated photodynamic therapy (PDT) effects [109]. Their research showed a twofold stronger inhibitory effect on gastric malignant cells of human compared to normal epithelial cells of stomach. Efficiency of Drug delivery in epithelial cells was notably higher than malignant cells. Ming et al. produced a multimodal composition involving CQDs coated with modified Fe3O4 on single-walled carbon nanotubes and subjected to NIR laser irradiation for PDT. This formulation, loaded with DOX and conjugated with an sgc8c aptamer for targeted imaging and therapy, showed potent effects in both photodynamic therapy (PDT) and chemotherapy [100].

Overall, Due to their low toxicity, CQDs are widely used in multimodal cancer treatment strategies. This approach leads to more effective inhibition of cancer cells, leveraging CQDs' capabilities in targeted therapy and enhancing treatment outcomes.

## Challenges in harnessing carbon dots for anticancer therapy

Despite multiple advantages to carbon dot-based therapy, like simple preparation method, low requirements for experimental equipment, stable photoluminescence properties, sensitive synthesis procedure, temperature-responsive characteristics, remarkable reversibility /recoverability, it also presents with a few drawbacks and challenges. The disadvantages include low yield, separation, poor control of particle size, severe synthesis conditions, etc. A few limitations of the carbon dot-based therapy are discussed in the following sub-sections.

### Biocompatibility and toxicity

Over the past decade, the rapid advancement in carbon dot production, development, and improvisation has widened the array of its potential applications, ranging from viable theragnostic agents to optical utilization like sensors, photocatalysts, information encryption, etc. Carbon dots abundantly exploit the versatility of carbon resulting in various favourable properties like high stability, multiple functional groups, eco-friendliness, biocompatibility, etc. [30, 110, 111]. Widely done in vitro cytotoxicity studies across diverse cell lines have unequivocally established the conspicuously low toxicity and high biocompatibility of CDs, even when administered at elevated concentrations [112]. Furthermore, to support the analysis, in vivo experiments affirm the safety profile of CDs, demonstrating their ease of excretion through the renal and/or hepatobiliary system [113]. CDs are fluorescent nanoprobes that exhibit effects in biological applications due to their excellent biocompatibility [94]. The toxicity of these CDs was assessed using standard mouse fibroblasts (NIH/3T3) in vitro [94]**.** The cytotoxicity and ability to track within cells and intracellularly are critical factors influenced by the chemical and surface functional charge. An elaborate study of vital organs, including the, heart, kidney, brain, lung, spleen, liver, bladder, and testicle in rats, via blood hematology and biochemistry analyses, resulted in an absence of noteworthy inflammatory responses. This collective evidence underscores the unequivocal safety of CDs for biomedical applications. Although the results are in favor of the implementation of CDs for theranostics purposes, the cell culture and animal models cannot replicate the complexity of human physiology [114].Henceforth, the lack of multi-model studies poses a great challenge to the clinical translation of a novel nanoparticle. Furthermore, the metabolism and excretion pathways of CDs are not fully understood, contributing to concerns about their toxicity in biological systems. Bioconjugation of QDs is common for drug delivery in cancer therapy; however, some CD materials are cytotoxic, posing challenges for effective cell delivery [94]**.** Similarly, the cytotoxic drug conjugates with CDs (although, results in increase in cytotoxicity of the cancerous cells due to the photothermal effect of the CD leading to an increase in reactive oxygen species (ROS) along with the drug effect [115–117] poses an increasing risk to the healthy cells due to lack of precision and specificity. Target specificity is rather an uninsured factor that restricts the long-term clinical use of CD.

The toxicity of CDs varies significantly depending on their surface modifications. According to a study, no in vitro toxicity was observed for any of the tested CDs, adverse effects were detected in vivo. Specifically, CD_3011, enriched with carboxyl groups on its surface, exhibited the highest overall toxicity and specifically affected the kidneys. This toxicity manifested with delayed effects, likely due to accumulation over time, as indicated by signs of gross toxicity, mortality, and changes in body weight dynamics [118]. On the other hand, CDN19, which also features surface enrichment with nitric groups, showed pronounced toxicity during the initial days of administration, reflected in changes in body weight dynamics and mortality rates [118]. However, mice appeared to adapt and recover subsequently, with no observable signs of toxicity based on histopathological and biochemical assessments at the study's conclusion.

Nevertheless, the findings emphasize that the toxicity of these CDs, particularly CD_3011 and CDN19 after repeated dosing, warrants careful consideration if they are to be utilized as a platform for drug development. Monitoring their effects over extended periods and evaluating potential accumulation-related toxicity are crucial steps in assessing their suitability and safety for biomedical applications.There are another study where Boron enriched CDs showed low cytotoxicity [119].

Developing biocompatible carbon quantum dots from sugarcane waste, showcasing their dual capabilities in nonlinear optics and antimicrobial activities. This approach not only addresses environmental sustainability but also opens new avenues for advanced materials in biomedical and technological applications [100]. In this context, a study revealed the fluorescent and biocompatible CDs derived from banana biomass using heat treatment. CDs are known for their beneficial physicochemical properties, making them highly desirable for bioimaging, biosensing, and disease detection [99].

By utilizing banana biomass, a renewable and sustainable resource, the study underscores an eco-friendly approach to producing C-dots. The heat treatment process facilitates the transformation of banana biomass into fluorescent C-dots that possess biocompatibility, ensuring minimal toxicity and suitability for biological applications [100, 120].

Overall, this research contributes to expanding the repertoire of carbon dots synthesized from agricultural waste, offering promising avenues for their use in advanced biomedical and diagnostic technologies. In a research, carbon dots were synthesized from tomato juice to harness the antioxidant capabilities inherent in this fruit. Additionally, the antioxidant potential was evaluated using the DPPH, and the cytotoxicity was assessed on A549 lung carcinoma cell lines [121, 122]. Importantly, these carbon dots demonstrated low toxicity towards healthy cells over a 72-h period, while inducing some cytotoxic effects in A549 cells [121].

Overall, the study highlights the potential of tomato-derived carbon dots as effective antioxidants with promising biomedical applications, while emphasizing their biocompatibility and low toxicity profile in healthy cell models. However, there are growing concerns about the toxicity of QDs to biological systems [123, 124].

Researchers are looking for substitute materials, such as CDs made from biowaste to allay these safety concerns. The synthesis of CDs from natural resources using hydrothermal carbonization methods has gained attention due to its straightforward process and minimal environmental impact [94]. These alternatives present advantages such as biodegradability, biocompatibility, and abundant availability, potentially resolving toxicity issues associated with conventional quantum dots (QDs). However, regulatory information regarding the biomedical use of QDs and CDs remains limited [94].

Carbon-based nanodots, in particular, exhibit enhanced imaging sensitivity, suggesting they could serve as safer alternatives for imaging and therapeutic purposes. Surface chemistry significantly influences their performance in imaging and biological applications [94, 125].

Nanoparticles have undergone three generations of development to enhance their biomedical applications. First-generation NPs faced challenges such as rapid clearance and accumulation in organs. Second-generation NPs introduced stealth coatings like polyethylene glycol (PEG) to evade immune detection and improve solubility [94, 99, 108]. Despite these advancements, the Enhanced Permeability and Retention (EPR) effect, which enables NPs to accumulate in tumors due to leaky vasculature, varies widely depending on tumor type and stage. Third-generation NPs are actively targeted using ligands such as proteins and nucleic acids, although this modification can affect their fluorescence properties and requires careful consideration. Overall, while QDs and CDs hold great promise in biomedicine, including applications in cancer imaging and therapy, addressing their safety profiles, optimizing biocompatibility, and elucidating their regulatory status are essential for their widespread adoption in clinical settings [94].

### Targeting specific *cancer* types

A variety of CDs have found extensive application in imaging cells, microorganisms, and plant tissue. In the realm of cellular and tissue imaging, CDs have demonstrated remarkable versatility. They showcase an adept ability to enter cells through diverse mechanisms, including temperature-dependent as well as energy-dependent micropinocytosis [126, 127]. Once inside cells, CDs disperse into various organelles, such as Golgi apparatus, mitochondria, endoplasmic reticulum, lysosomes, and nucleolus. This distribution is contingent upon the distinctive nanostructures of CDs and the specific cell types involved. The imaging of organelles, notably mitochondria and nucleolus, provides invaluable insights into the study of diseases associated with these cellular components, including cancer, diabetes, Parkinson's disease, Alzheimer's disease, and cardiac dysfunction [128]**.** As an example, studies have successfully demonstrated the nucleus-targeted imaging capabilities of CDs derived from mPD and L-cysteine in both dead and living cells [129]. Moreover, when these carbon dots are coupled with protoporphyrin IX, they gain nucleus-targeted photodynamic therapy (PDT) capabilities, showcasing efficacy in tumor ablation without inducing toxicity after laser irradiation. Similarly, the application of carbon dots sourced from Lactobacillus plantarum as staining agents has proven useful in imaging biofilm-encased microorganisms. This imaging technique provides valuable insights into the physiological and morphological state of bacteria within biofilms. Apart from imaging, phototherapy can also be utilized as non-invasive therapeutic strategy involving photothermal therapy (PTT) and photodynamic therapy (PDT). This strategy converts emitted light into reactive oxygen species and heat through photosensitizers, inducing localized apoptosis in cancer cells. Carbon dots have attracted significant interest as potential agents for phototherapy owing to their distinctive optical characteristics, excellent water solubility, and durable photostability. Nevertheless, a hurdle emerges in photodynamic therapy because of the hypoxic tumor microenvironment and the swift exhaustion of oxygen during the procedure, constraining the therapeutic effectiveness of carbon dots. In response to this challenge, researchers like [114] have introduced nanocomposites that incorporate CDs decorated with C_3_N_4_. This innovative approach, ultimately addressing hypoxia-induced resistance to PDT and inhibiting tumor metastasis.

### Drug delivery applications

In addition to their role in advanced anticancer phototherapies, CDs exhibit a remarkable capacity to synergize imaging tools with genes or drugs, creating sophisticated imaging-guided nanohybrids. This integration aims not only to enhance delivery efficiency but also to provide nuanced advantages in therapeutic strategies. Within the realm of drug delivery, a pivotal facet of safe and effective treatment revolves around the precise transport of medicine to designated locations in the body and its controlled release over time. The paramount importance of the meticulous selectivity inherent in drug delivery systems and controlled drug release cannot be overstated. This becomes indispensable for the comprehensive evaluation of the therapeutic efficacy of medicines [130, 131]. Deep red emissive CDs with multiple paired amino groups and α-carboxyl can be designed to elevate the precision of drug delivery performance and tumor-specific imaging [132]. This strategic design enabled these CQDs to selectively target tumors, including glioma [128, 133].

### Regulatory and safety considerations

The widespread popularity of CDs in energy, biomedical, and optical fields can be attributed to their environmentally friendly, facile, and diverse synthetic methods. Coupled with their excellent electrical and optical properties, good biocompatibility, and low cost, CDs have become pivotal in scientific exploration. Despite notable progress in the study of CDs, there remain critical challenges, particularly in the absence of a scalable and systematic synthesis protocol. A fundamental issue is the lack of a clear understanding of their reaction mechanisms and nucleation processes. To address this, a systematic exploration of the effects of reaction conditions and precursors on CD performance.The photoluminescence (PL) mechanism of CDs remains a topic of debate, with differing conditions and precursors contributing to varied PL behaviors [134, 135].

Moving forward, future research on CDs should not only delve into the PL mechanism and synthetic methods but also focus on designing CD structures tailored to specific applications. In the realm of sensing, efforts should be directed towards developing functional CDs with stability, selectivity, sensitivity, and high quantum yield. For applications in data security and anticounterfeiting, improvements in the lifetime, stability of CDs-based afterglow materials, and quantum yield, are essential. Additionally, exploring multitechnology approaches for security encryption may be the future trend. In the energy sector, where CD applications are still in their infancy, key scientific issues need attention [136]. These endeavors are crucial for shedding light on the energy storage mechanisms and electrochemical performance of CDs-based materials.

### Patents on carbon dots

The landscape of patents is constantly evolving, especially in the realm of gene delivery for combating cancer using nanocarriers. Table 1 outlines several patents focused on the CDs synthesis [137]. Specifically, the invention employs an ultrasonic-assisted wet-chemical process to produce distinctive carbon dots from low-quality Indian coals. This method ensures the carbon dots possess specific characteristics and are size-controlled. Moreover, the invention emphasizes its simplicity and environmentally friendly nature, offering a straightforward approach to fabricating these carbon dots. This innovation not only enhances the utilization of low-quality coal resources but also provides a sustainable method for generating carbon dots with tailored properties suitable for various applications [137]. Another invention was revolved around a novel drug delivery system known as the quantum dot drug delivery system, which utilized quantum dot nanoparticles that responded to light stimuli for controlled drug release. These quantum dots were water-soluble and specifically engineered to absorb light at a particular wavelength [138].

**Table 1:** Recent Patents on carbon dots

Serial no	Patent no	Classification	Patent /publication title	Inventors	Organization	Issue/publication date	References
1	US10655061B2	Based on the preparation of Carbon dots	The method used to prepare blue-flourescence emitting carbon dots (CDTS) from Indian coals that are high in sulfur and sub-bituminous tertiary	Saikia et al	Council of Scientific & Industrial Research, New Delhi, Delhi	May 19, 2020	[137]
2	WO 2018167618 A	Based on type of NP: Quantum Dot	Light Responsive Quantum Dot Drug Delivery System	Naasani I	Nanoco Technologies Ltd., GB	Sept. 20, 2018	[138]
3	WO2019232701A1	Based on curcumin quantum dots	Curcumin carbon quantum dots and use thereof	Chih-Ching HuangChin-Jung LinShiow-Yi CHENLung CHANGHan-Jia Lin	Huang Chih Ching	Dec.12.2019	[139]
4	CN108837156B	Based on Carbon dots	Preparation method of carbon dot drug-loading system	耿丽娜张泽帝段相林常彦忠	Hebei Normal University	Aug.17,2021	[140]
5	US 8,535,726 B2	Based on type of NP: Carbon	Supramolecular Functionalization of Graphitic Nanoparticles for Drug Delivery	Dai H, Liu Z, Li X, Sun X	The Board of Trustees of the Leland Stanford Junior University, US	Sep. 17, 2013	[138]

In practical terms, the drug molecules were encapsulated or conjugated with the quantum dots. When exposed to light of the appropriate wavelength, the quantum dots undergo a process that triggers the release of the drug molecules. This mechanism allows for precise and controlled drug delivery to targeted tissues or cells within the body.

The advantages of this technology include enhanced specificity and efficiency in drug delivery, minimizing side effects by ensuring that the drugs are released only at the desired location upon exposure to light. This approach can potentially revolutionize therapies where spatial and temporal control over drug release is critical, such as in cancer treatment, localized infections, or other targeted therapies.

The method described for preparing these quantum dot drug delivery systems ensures that the process is reproducible and scalable, making it suitable for clinical applications. Overall, the invention represents a significant advancement in the field of nanoparticle engineering and targeted drug delivery systems.

According to a new invention, which was within the field of nanoparticle engineering, specifically focused on an engineered nanoparticle containing curcumin carbon quantum dots and their application for antiviral purposes [139]. Additionally, the invention encompasses a method for preparing these curcumin nanoparticles. There was also a patent study, which described a method for preparing a carbon dot drug-loading system using peony pollen as a raw material and urea as a modification reagent. Carbon dots of uniform particle size were synthesized via a hydrothermal process. These carbon dots undergo light-shielding coupling reactions separately with doxorubicin hydrochloride (DOX) and ferric citrate (FAC), resulting in CDs-DOX and CDs-FAC, respectively.

CDs-DOX prepared by this method exhibit high drug loading, slow release characteristics, and targeted release to cancer cells. CDs-FAC show high drug loading, slow release in gastric juice compared to intestinal juice, reduced stomach irritation from FAC, enhanced absorption by the small intestine, and suitability for oral administration. The preparation method is straightforward, offering simple and convenient operations with easily controlled reactions.

The resulting products effectively enhance the therapeutic efficacy of individual DOX or FAC treatments, mitigate their toxic and side effects, and are applicable for treating diseases associated with iron disorders. The active agent (drug) carried by these nanoparticles can be released in the cellular environment.

This innovative approach allows particularly cancer therapy where tumor-specific conditions can trigger enhanced drug release. The method enhances drug efficacy while potentially reducing side effects by ensuring precise delivery and release mechanisms within the cellular environment.

## Opportunities for advancing carbon dot-based anticancer strategies

The timely detection of cancer cells is pivotal for enhancing the efficacy of treatment. There exists a substantial need for inventive drugs and methodologies that can precisely and effectively target cancer. A recent revolutionary advancement in cancer treatment, termed "Nanotheranostics," combines both cancer diagnosis and therapy into a cohesive platform. This integrated strategy enables the acquisition of highly specific data, facilitating accurate, highly sensitive, and minimally disruptive cancer treatments.

A broad spectrum of nanotheranostic agents is accessible, utilizing combinations of two or more imaging techniques. Additionally, various therapeutic modalities are integrated, encompassing a comprehensive approach involving PDT/PTT/CHT [94]. While these methods have gained widespread popularity, it's important to note that each type of nanotheranostic agent possesses specific characteristics and inherent limitations [141].

**Fig. 5:**
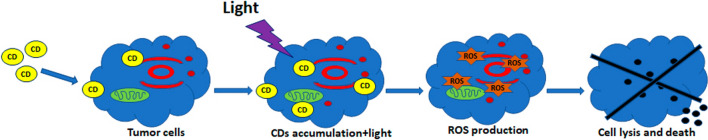
Illustration of Photodynamic Therapy: CDs infiltrate the cell membrane and amass in cytosol. When exposed to light irradiation, CDs are activated, triggering the generation of reactive oxygen species (ROS). This process ultimately results in cell lysis and cell death. Reproduced with CC-BY L License from [70]

Various nanomaterials have been utilized in the realms of cancer detection, diagnosis, and treatment, with carbon dots (CDs) standing out for their distinctive properties. Noteworthy among these features are their low production cost, photostability, biocompatibility, unique optical and chemical properties, high specificity to targets, hydrophilicity, and non-toxic nature. These attributes collectively position CDs as promising elements for effective cancer detection [70]. Primarily composed of carbon, oxygen, and hydrogen, CDs have garnered significant attention as ideal nanocarriers for cancer-specific drug delivery owing to their compact size and robust conjugation capacity facilitated by surface functional groups like carboxyl and amino groups. CDs are acknowledged as viable choices for cancer diagnosis. [70]. Recent research underscores the extensive utilization of CDs in cancer cell imaging and inducing cell toxicity and causing irreversible damage to cancer cells. Previous studies have demonstrated the superior selectivity of PDT and PTT, leading to a significant reduction in systemic toxicity compared to conventional chemotherapy and radiotherapy (Fig. 5) [142]. Consequently, CDs with their distinctive photo-theranostic capabilities, exhibit substantial potential for application in tumor treatment. CDs derived from Simarouba glauca leaves exhibit anticancer properties against MCF-7 human breast cancer cells [143]. Additionally, the study unveils a novel fluorescent probe based on these CDs for the detection of doxycycline. The synthesized CDs demonstrate remarkable stability and emit blue fluorescence.The primary aim of this section is to underscore the possibilities of CDs as nanotheranostic agents in anticancer applications, focusing on their use in bio-imaging cancer cells, nanotheranostics, surface chemistry, and mechanisms of action.

According to [97] two commonly used bottom-up techniques were used to create glucose-derived CDs: hydrothermal carbonization (HT) and microwave-assisted (MW). A comprehensive analysis was conducted to revealing distinct subtypes based on the synthesis route. Notably, CDs produced through the HT method showcasing significant therapeutic potential. These CDs displayed high biocompatibility and promising prospects for in vitro and in vivo applications.

P-dots displayed remarkable luminescence properties, making them well-suited for bioimaging applications [144]. Additionally, they demonstrated significant anticancer activity, which was attributed to caspase-3-related cell apoptosis. The carbon dots derived from procaine synthesis showcased a dual functionality, encompassing both bioimaging and anticancer activity. This dual capability suggests their potential as safe and effective clinical nanotherapeutics.

### Surface engineering and functionalization

Various carbon nanostructures, including graphene, fullerene, carbon nanotubes, and carbon nanodots (CDs), showcase unique characteristics like robust fluorescence and photo-stability. Also its chemical properties, lesser toxicity, and biological compatibility making it more feasible for bio-engineering process. Over the past few years, numerous synthesis routes for CDs have been developed, prompting a demand for comprehensive research to enhance their functionality. In this context, two primary approaches, doping and surface functionalization stand out as major methods to regulate the chemical, optical, and electrical properties of CDs [70, 145–150]**.**

CDs represent a category of zero-dimensional semiconductor nanocrystals, characterized by their roughly spherical shape and diameter of less than 10 nm. These nanoclusters are composed of a small number of molecules or atoms [145]. Typically, quantum dots have substantial particle sizes and molecular weights that can reach hundreds of thousands. In contrast, carbon dots (CDs) are characterized by smaller particle sizes, typically in the range of a few nanometers, and molecular weights ranging from a few thousand to tens of thousands. Their elemental composition usually includes carbon, hydrogen, oxygen, and nitrogen, presenting a marked difference from untreated, nonfluorescent carbon particles [151].

**Fig. 6:**
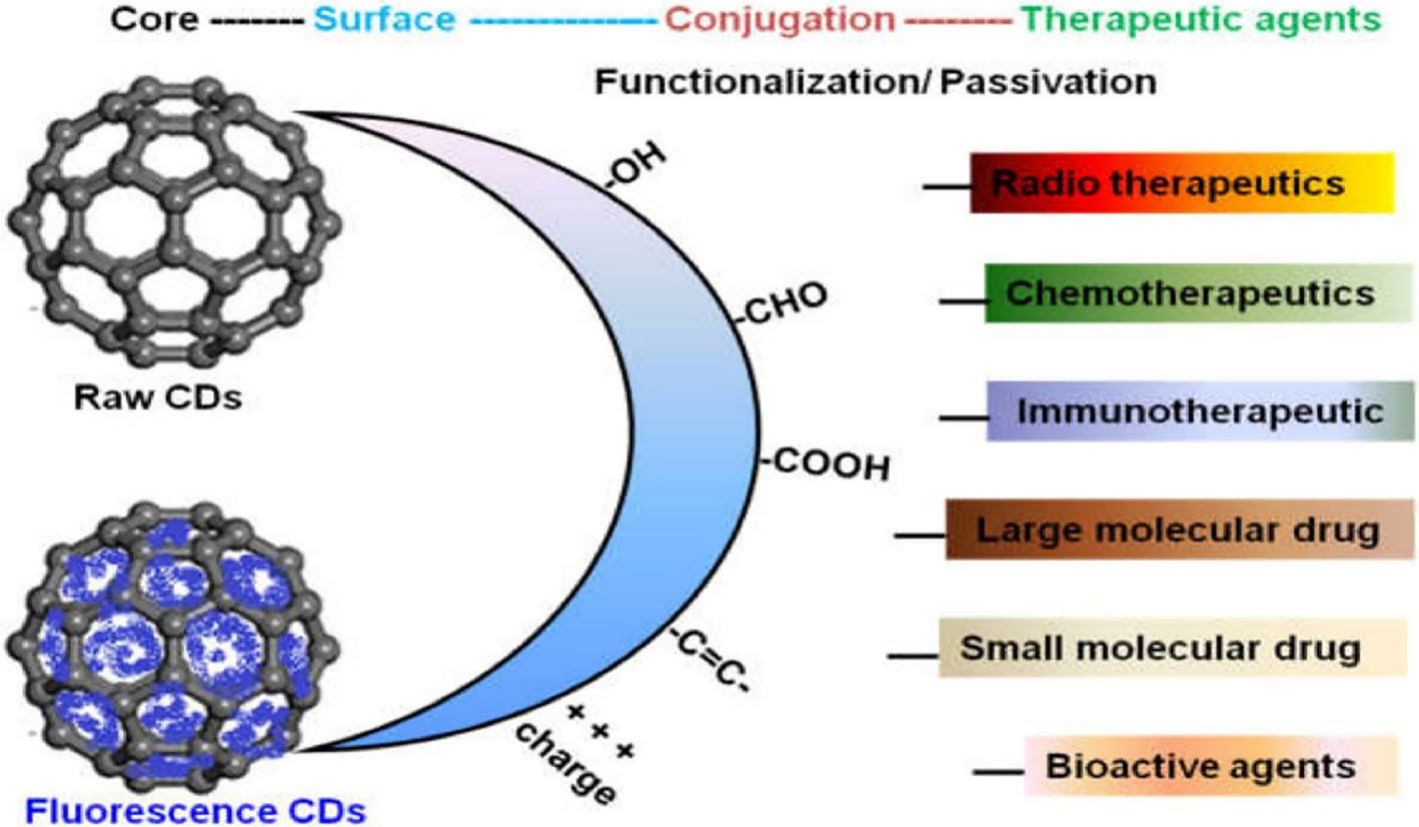
Surface-modified CDs are crafted for enhancing functionality in therapeutic and diagnostic applications. Reproduced with the CC BY License from [18]

The unique structural features of CDs confer excellent physical and chemical properties (Fig. 6). While previous literature has explored several applications of CDs primarily based on chemical engineering, there is limited research reported on the surface engineering of CDs. Acting as structural regulators, CDs can induce the formation of materials into spheres, octapods, and octahedrons. Conversely, there are instances where the structural morphology and characteristics of the material remain unchanged after compounding with CDs. CDs play crucial roles as structural regulators, acting as surfactants, selectively adsorbing, serving as reducing agents, and inducing crystallization [152–154].

Furthermore, CDs with surface-active chemical groups exhibit excellent surface functions and are employed as chemical additives. The functional groups on the surface of CDs contribute to an adsorption effect, forming a thick lubricating film on friction surfaces to enhance friction resistance [147]. The combination of these functional groups and polymer chains on the film surface can merge to create a protective layer of film [147, 152, 155–158].

According to Chowdhury et al., the inclusion of biotin on the surface of riboflavin-tethered carbon dots is crucial for selectively targeting cancer cells over normal cells. Under UV light conditions (340–420 nm), RCD1s exhibited no significant impact on the viability of both cancer and normal cells. Surface functionalization using specific inorganic, organic, biological, or polymeric materials represents a practical approach to tailor the emission properties of carbon dots (CDs) [147, 159]. Generally, CDs are considered to have a core–shell structure comprising a carbon core with surface passivation by various functional groups like carboxyl, hydroxyl, and amine. These surface functional groups not only facilitate the generation of fluorescence (FL) through surface defects but also provide reactive sites [157]. Due to their convenient conjugation procedures, amines and carboxyls are the most common reactive sites on CDs for subsequent surface functionalization [146]. CDs synthesized from PEG containing larger molecular weight with slightly larger particle size exhibit almost identical morphology.

A similar phenomenon is observed in the case of N-atom doping. Amino-terminated CDs have also been prepared through the surface functionalization of CDs with EDA via a hydrothermal approach [160]. The surface functionalization process involves multiple steps, and a well-designed conjugating scheme is necessary to meet specific requirements. Typically, three established conjugation strategies for surface functionalizing CDs include covalent bonds, hydrogen bonds, and electrostatic interactions. Among these, covalent bonds are stronger than other intermolecular forces, making covalent modification crucial for achieving better control over the size, shape, and physical properties of CDs.

The surface engineering of carbon dots (CDs) through covalent bonds commonly involves the use of the classical catalyst couple, ethyl(dimethylaminopropyl) carbodiimide (EDC), and N-hydroxysuccinimide (NHS), based on carbodiimide chemistry. This method is particularly favored due to the abundant presence of amino and carboxyl groups in biomolecules. According to Zhang et al., the derivatives of quinoline and cyclic DTPA dianhydride (cDTPAA) function individually as specific zinc probes, with quinoline-derived functionalized CDs transforming into a zinc-specific receptor. This transformation is characterized by a change in fluorescence color from blue to green upon zinc ion activation at a wavelength of 350 nm [161].

In another study by Ye and colleagues, CD surfaces was achieved through the reaction between amino and sulfonyl chloride groups [162]. Additionally, surface-initiated atom transfer radical polymerization (ATRP) has proven effective in surface engineering CDs for creating a poly(N,N-dimethylaminoethyl methacrylate) (PDMA)-functionalized CD-based platform. In this process, amino-terminated CDs were converted to Br-terminated CDs through a reaction with 2-bromoisobutyric acid activated by N,N0-carbonyldiimidazole. The resulting Br-terminated CDs were then mixed with 2-(dimethylamino) ethyl methacrylate (DMAEMA) and NaCl in a degassed ethanol suspension of CuCl, allowing for a reaction at room temperature for 3 h.

Hydrogen bonds and electrostatic interactions, however weaker than covalent bonds, are nonetheless appropriate for the surface engineering of CDs. Utilizing the electrostatic interactions between negatively charged gold nanoparticles (AuNPs) and positively charged CDs, a dual-mode nanosensor was created to distinguish glutathione (GSH) from Cys/Hcy. The colour shift of AuNPs and Förster resonance energy transfer (FRET), two distance-dependent processes, served as the basis for this differentiation [163].

### Combination therapies

CDs represent a novel class of zero-dimensional carbonaceous nanomaterials boasting compelling biomedical properties. Consequently, CDs find diverse applications as sensing, bioimaging, and drug delivery. Recent advancements have uncovered the potential of CDs as effective photosensitizers capable of generating reactive oxygen species under light excitation. Additionally, they can serve as photothermal agents, converting light energy into heat. This dual functionality positions CDs for application in photodynamic therapy and photothermal therapy for cancer treatment.

Nanotheranostics, which combine diagnostic and therapeutic functions in a single platform, have shown significant promise in cancer treatment. Among the materials explored for nanotheranostic applications CQDs, CNDs, and other carbon-based nanoparticles have gained considerable attention due to their unique properties[164]. CQDs are small, fluorescent carbon nanoparticles with sizes typically below 10 nm (Table 2) [10, 94]. They exhibit excellent photostability, biocompatibility, and tunable fluorescence properties, making them ideal for bioimaging and photodynamic therapy (PDT) applications. CQDs can be synthesized from various carbon sources, including amino acids and proteins, which enhance their biocompatibility and functional versatility [164]. Furthermore, CQDs have shown potential in photothermal therapy (PTT) and as carriers for drug delivery, providing a comprehensive approach to cancer treatment [99, 108]. Carbon Nano Dots (CNDs) (Table 2), often used interchangeably with CQDs, are another class of carbon-based nanoparticles. They share similar optical properties with CQDs but differ slightly in their synthesis methods and surface functionalities. Their ability to be functionalized with different biomolecules enhances their targeting capabilities, making them effective in cancer theranostics [164]. Carbon-Based Nanoparticles Beyond CQDs and CNDs, other carbon-based nanoparticles such as GQDs and fullerenes (Table 2) have been explored for nanotheranostics. GQDs, for example, offer excellent electrical conductivity, high surface area, and strong fluorescence, making them effective in bioimaging and as drug delivery systems[100]. Fullerenes, with their unique cage-like structure, have been studied for their photodynamic and photothermal properties, providing another avenue for cancer treatment. CQDs and CNDs can be synthesized from a wide range of precursors, including organic materials, which can be tailored to enhance biocompatibility and functionality—GQDs and fullerenes require more complex synthesis processes but offer unique properties such as higher electrical conductivity and larger surface area. CQDs and CNDs exhibit strong fluorescence and photostability, which are advantageous for imaging applications.GQDs also offer strong fluorescence and can be used in combination with other nanoparticles to enhance imaging and therapeutic efficacy. CQDs and CNDs generally demonstrate low toxicity and high biocompatibility, which are crucial for clinical applications.The biocompatibility of GQDs and fullerenes varies depending on their functionalization and synthesis methods, necessitating thorough evaluation for biomedical use.CQDs and CNDs are effective in PDT and PTT due to their strong light absorption and conversion properties.GQDs can be utilized in combination therapies, integrating PTT, PDT, and drug delivery. In conclusion, CQDs, CNDs, and other carbon-based nanoparticles hold significant promise in nanotheranostics for cancer treatment. Their unique optical properties, biocompatibility, and multifunctional capabilities make them versatile tools for simultaneous diagnosis and therapy, offering a comprehensive approach to combating cancer [10, 94].

**Table 2:** Comparision of carbon quantum dots,carbon nano dots and GQDs and fullerenes

Feature	Carbon Quantum Dots (CQDs)	Carbon Nano Dots (CNDs)	Other Carbon-Based Nanoparticles (GQDs, Fullerenes)	References
Size	Typically below 10 nm	Generally less than 10 nm	GQDs: typically < 10 nm; Fullerenes: variable, often < 1 nm in diameter	[10, 94]
Optical Properties	Excellent photostability and tunable fluorescence properties	Similar optical properties to CQDs, strong fluorescence	GQDs: strong fluorescence and excellent electrical conductivity; Fullerenes: unique photodynamic and photothermal properties	[100, 164]
Biocompatibility	High biocompatibility, enhanced by synthesis from carbon sources like amino acids and proteins	High biocompatibility, high aqueous solubility, robust chemical inertness	GQDs: variable biocompatibility depending on functionalization; Fullerenes: biocompatibility varies, requiring thorough evaluation	[94]
Synthesis Sources	Various carbon sources, including amino acids and proteins	Organic materials, synthesis methods differ slightly from CQDs	GQDs: complex synthesis processes involving top-down or bottom-up methods; Fullerenes: complex synthesis involving arc discharge, laser ablation, or chemical vapor deposition	[94]
Functional Versatility	Enhanced by diverse synthesis sources, highly tunable for various applications	Functionalized with different biomolecules, enhancing targeting capabilities	GQDs: high surface area and conductivity, useful in combination therapies; Fullerenes: cage-like structure suitable for functionalization and therapeutic applications	[99, 108]
Imaging Techniques	Beneficial for multimodal imaging processes like photoacoustic and fluorescence imaging—MRI, or magnetic resonance imaging	Suitable for bioimaging applications	GQDs: effective in bioimaging and combined with other nanoparticles for enhanced imaging; Fullerenes: studied for photodynamic and photothermal properties, useful in imaging	[99, 108]
Therapeutic Applications	- Photodynamic Therapy (PDT)—Photothermal Therapy (PTT)—Drug delivery carriers, providing a comprehensive approach to cancer treatment	- Targeted drug delivery—Gene delivery—Effective in cancer theranostics	GQDs: utilized in combination therapies (PTT, PDT, drug delivery); Fullerenes: potential for photodynamic and photothermal therapy, offering another avenue for cancer treatment	[99, 108]

### Imaging and diagnostics

**Fig. 7:**
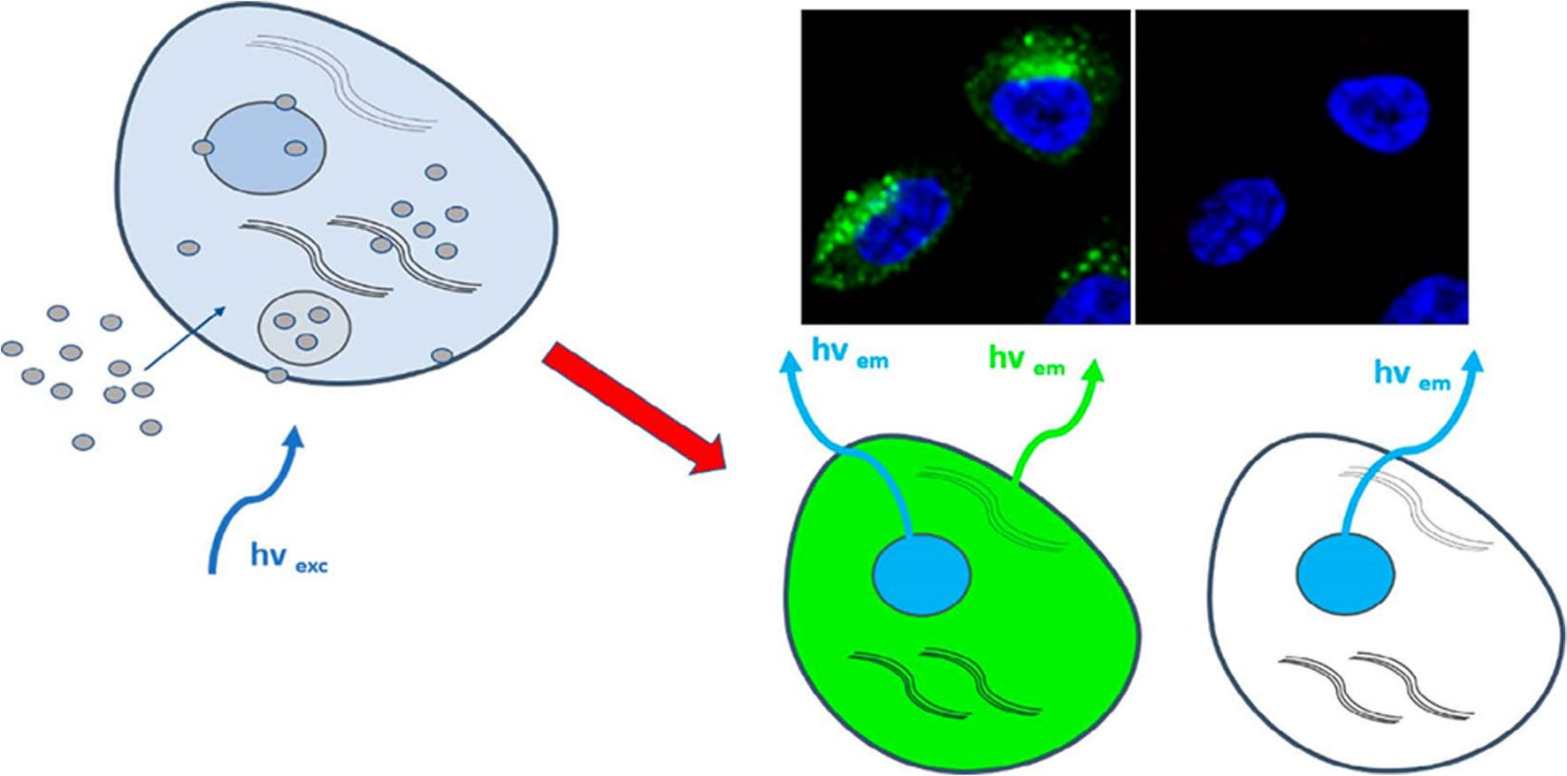
Visualization of CDs Cellular Uptake for Bioimaging: The left segment portrays a cell sketch, showcasing CDs penetrating the cell membrane and amassing in the cytosol (represented by gray circles on the left). On the right, there is an illustration of the characteristic fluorescent green staining attributed to intracellular CDs, with DAPI-stained cell nuclei (in blue) visible in the image. Reproduced with the CC BY License from [70]

CDs have exhibited a significant role for both in vitro and in vivo imaging. Given their physical–chemical attributes and size, CDs can readily traverse biological membranes and gather in the cell cytosol or nucleus, serving effectively as a fluorescent probe (Fig. 7).

Bioimaging plays a pivotal role in comprehending the structural organisation of cells. CDs is gaining increasing attention with optical contrast between the imaged region and the surrounding background [70, 165]. Traditional imaging often relies on wavelengths, but CDs face challenges due to significant background interference caused by the limited penetration of light in tissues. Up-conversion photoluminescence (UCPL) proves beneficial in this regard [70].

As per Wang et al., the biomass as carbon source has led to the development emission of deep-red CDs with excellent UCPL for simultaneous cellular and two-photon imaging. Additionally, CDs with multiphoton photoluminescence emission have been designed for cellular bioimaging. Two-photon imaging using CDs is applicable both in vitro and in vivo [146, 150, 166].

While there are limited dyes available for living cell imaging, especially as fluorescence microscopy typically requires cell fixation [70, 167].

Jiang's group synthesized red, green, and blue (RGB) photoluminescent CDs, assessing their cytocompatibility and cell imaging. The study demonstrated cell viability over 90% in MCF-7 human breast cancer cells incubated with the three types of CDs, with living cells predominantly fluorescing in the cytoplasm, indicating CD penetration through the cell membrane [168].

Several studies on different cell lines, including HeLa, SMMC-7721, and HEK 293, confirmed the biocompatibility and non-cytotoxic nature of CDs at higher concentrations [167]. CDs were found to enter cells, primarily localizing in lysosomes/endosomes [168–170]. Reports also exist on naturally sourced carbon dots (CDs) that exhibit non-cytotoxic behavior and efficiently penetrate cells during the incubation period. These naturally sourced CDs demonstrated blue-green fluorescence in the cytoplasm without affecting the nuclei [171].

Extensive research has been conducted on the potential use of fluorescent CDs in vivo for biomedical applications. In a study by Yang et al., CDs exhibited strong fluorescence even after intradermal, subcutaneous, and intravenous injection, proving to be biocompatible and non-toxic [93]. Similar findings were observed in the study by Huang et al., which evaluated the effects of three different injection methods of fluorescent CDs on blood circulation [172]. Notably, CDs were efficiently and rapidly taken up by tumors when administered subcutaneously [162, 173].

Several biodistribution studies using radiolabeled, photoluminescent CDs for in vivo NIRF imaging and positron emission tomography (PET) have shown that CDs are gradually eliminated through renal and fecal pathways without causing obvious toxic effects on animals [146, 174]. The findings from both in vitro and in vivo studies are promising for the application of fluorescent CDs in cancer diagnosis through imaging. Recent reports highlight the capability of CDs to recognize tumor cells with specific cell targets [175].

**Fig. 8:**
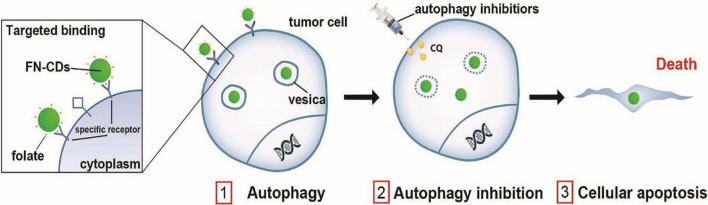
Illustration of Targeted Tumor Visualization and Therapy: FN-CDs are directed towards tumor cells through specific binding with membrane receptors facilitated by folate acid. Subsequently, they persist in a stable state within autophagic vacuoles in the cytoplasm. Upon the application of autophagy inhibitors, FN-CDs are released from the autophagic vacuoles, leading to the initiation of cell death through the apoptotic signaling pathway. Reproduced with the CCBY License from [131]

In a study by Song et al. [176] when a mixture of NIH-3T3 and HeLa cells was cultured and analyzed, after a 6-h incubation, only HeLa cells exhibited bright fluorescence with C-dots-FA, indicating selectivity for FR-positive cancer cells. Li and colleagues created a novel autophagy-regulated targeted tumor therapeutic approach by mixing biocompatible N-doped carbon dots (N-CDs) with folic acid (FA) (FN-CDs). FN-CDs exhibited a wide range of high-targeting ability (26 types of tumor cell lines) and influenced cellular metabolism leading to autophagy (Fig. 8) [131].

**Fig. 9:**
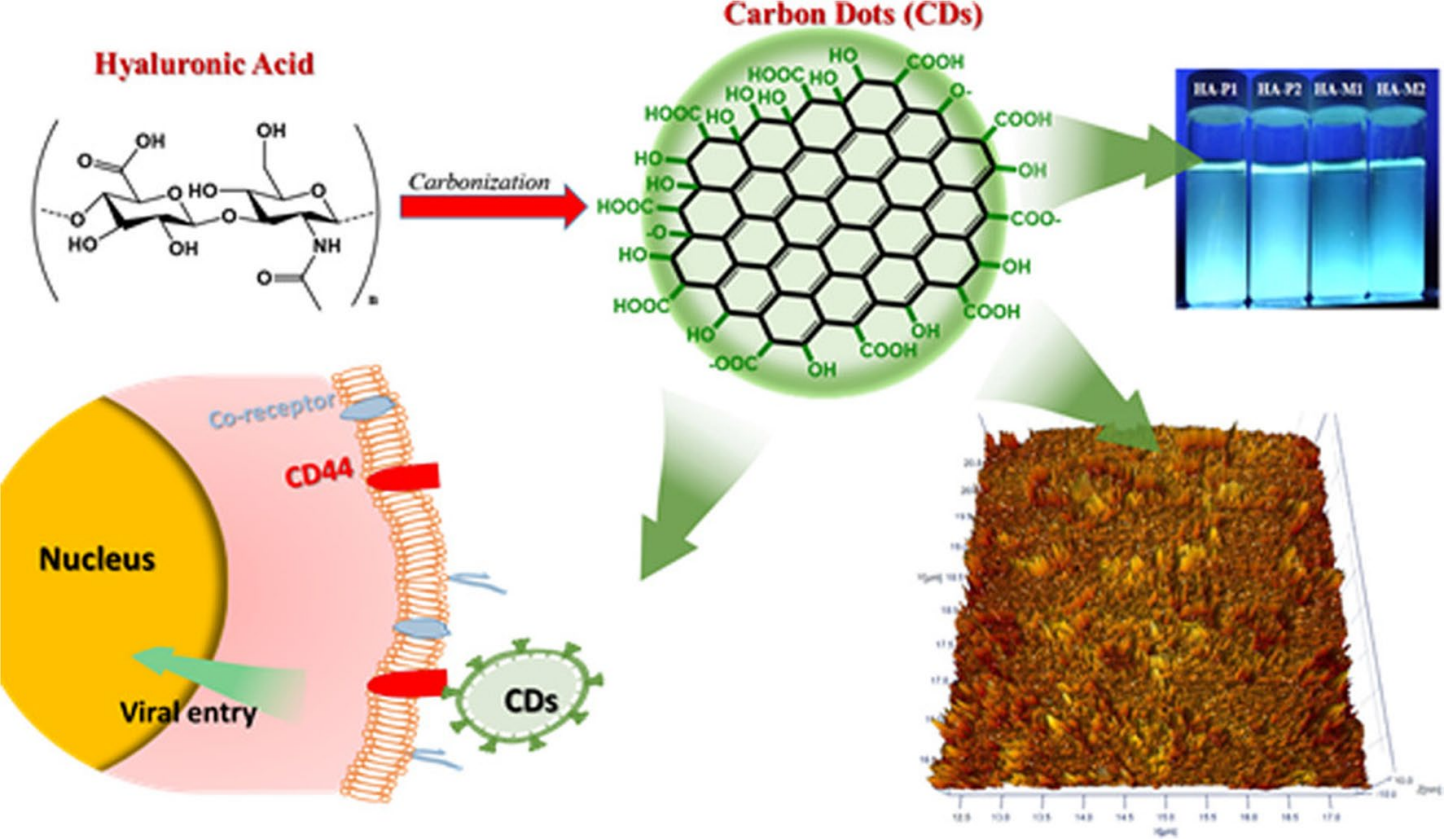
Schematic diagram of production of HA CDs and their mechanism on HeLa cancer cells. Reproduced with the CC BY license from [177]

**Fig. 10:**
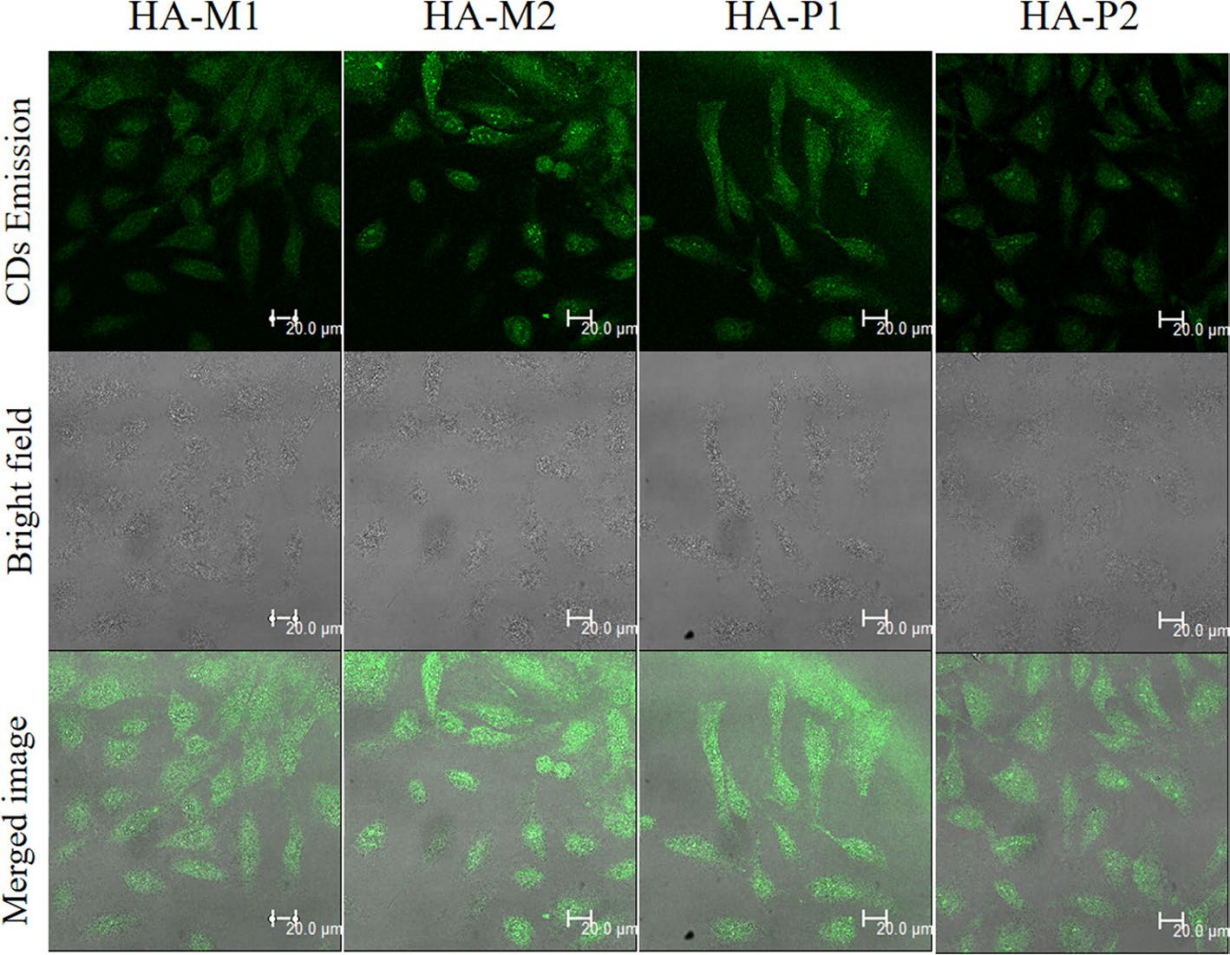
Images of CLSM of HeLa cells after incubation period of one hour with HA-based CDs by excitation at 488 nm. The scale bars represent 20 μm. Reproduced with the CC BY license from  [177]

Aung et al. investigated CDs from hyaluronic acid as bioimaging agents for cancer cells [177]. The effects of the synthesis times 2 min and 2 h, using both methods on CD characteristics are thoroughly examined. The resulting CDs displayed average diameters with maximum absorption wavelengths of 234 nm (HA-P1), 238 nm (HA-P2), 221 nm (HA-M1), and 217 nm (HA-M2) [177]. The quantum yields were determined to be 12% for HA-M1, 7% for HA-M2, 9% for HA-P1, and 23% for HA-P2. Cytotoxicity and in vitro activity were assessed using a cell counting kit-8 assay and confocal laser scanning microscopy, showing low toxicity with cell viability above 80% (Fig. 9 and Fig. 10).

According to a study, IndiFluors represent a significant advancement in the design of full-visible color-tunable fluorophores for bioimaging. Their unique donor–acceptor–donor system allows for precise control over their optical properties, while their structural stability and minimal cytotoxicity make them highly effective for live cell imaging. The specific application of PM-Mito-OH demonstrates the practical benefits of IndiFluors in monitoring mitochondrial health and function, showcasing their potential for broad use in biomedical research [178].

### Personalized medicine approaches

Chemotherapy, radiation therapy, and surgery are limited in their ability to treat cancer since there isn't a single treatment plan that works for every kind of cancer [179].

The goal of tumour theranostics is to create novel, biocompatible, and biodegradable structures that can effectively deliver target therapy. Many theranostic nanoparticle varieties have been made recently, but not all of them are effective and secure for use in cancer treatments [180]. In this regard, because of their previously mentioned physicochemical and optical characteristics, carbon dots are thought to be promising candidates for creating tumour theranostic therapy.

In fact, CDs have a number of benefits over other theranostic agents now on the market, including minimal cytotoxicity, biocompatibility, and stable photoluminescence. These advantages are compared to those of lipidosomes, polyester micelles, and metal–organic framework (MOF)-based nanomedicine [181]. As a result, CDs are now acknowledged as useful tumour theranostic drugs for tumour therapy and in vivo imaging as shown in Table 3.

**Table 3:** Complexes of carbon dots and their biological activity in cancer

Conjugates-CDs	Activity of CDs-conjugates	References
CDs-HA (hyaluronic acid)	Keeps HA functioning: HA has a strong affinity for CD44 receptors, which are overexpressed on the surfaces of many cancer cells	[98]
CDs-MnFe2O4/DOX-4	Function as a ligand that targets cancer cells specifically. Useful fluorescent markers for in vivo research. Additionally, it demonstrated how DOX is released under controlled conditions at low pH	[182]
PEG-Citosan nanogels loaded with CDs-DOX	High therapeutic efficacy achieved by combining chemotherapy and photothermal therapy in a synergistic way	[183]
FA: flolic acid/DOX-CDs	The fluorescent signal-based drug release monitoring in real-time is made possible by the system	[184]
CDs-DOX/biodegradable charged polyester vectors (BCPVs)/siRNA	The release of DOX from complexes was caused by the heat-generated enhancement of the nanocomplexe's anticancer activity	[185]
CDs-PEGFA/ZnPc (Zinc Phthalocyanine) or R780 iodide or Ce6	By observing the blue and red fluorescence of CDs, the targeting of ZnPc/CDs-PEGFA was assessed. Additionally, the HeLa cells' PDT was shown	[186]

## Economic feasibility and scalability of implementing carbon dots in mainstream cancer therapy practices

Implementing CDs in mainstream cancer therapy practices is an exciting and promising field, but It has a number of issues that must be resolved for it to be economically viable and scalable [187]. CDs can be synthesized through various methods like chemical oxidation, hydrothermal/solvothermal synthesis, and electrochemical synthesis. Despite the diversity in synthesis methods for CDs, there remains a significant demand for simple and cost-effective approaches. The current production costs are relatively high due to the complexity of these processes and the need for high-purity materials and controlled environments [187]. Transitioning from laboratory-scale to industrial-scale production requires significant investment in infrastructure and technology. Ensuring consistency and quality of CDs at large scales is crucial and challenging. Advancements in synthesis techniques that reduce costs and enhance scalability are necessary. Development of automated and high-throughput synthesis systems could reduce labor and operational costs. Significant initial investment is required for research, development, and clinical trials. Coal is a particularly affordable carbon source among those that are accessible. It is naturally abundant material compared to other derivatives like hydrocarbons. Coal is a promising candidate for CD production because its carbon structure is more easily oxidatively displaced than pure sp2-carbon structures, making it an attractive carbon source for high-value CDs. Using coal as a source for synthesizing and converting coal into high-value, ecologically acceptable nanomaterials using (CQDs) is a viable and alternative process [187]. This approach leverages coal's abundance and cost-effectiveness, providing a sustainable option for CDs production. Compared to pure sp^2^-carbon structures, coal's carbon can be oxidatively shifted more easily hence making it a promising candidate for creating high-quality CDs. This method could not only enhance the value of coal but also contribute to the development of green nanotechnology.

The economic feasibility and scalability of implementing quantum-sized carbon dots in mainstream cancer therapy depend on overcoming significant challenges related to production costs, regulatory approval, clinical integration, and proving economic viability. However, there are numerous opportunities, particularly in advancing synthesis techniques, regulatory collaboration, and demonstrating clear clinical benefits. Strategic investments in research, partnerships, and infrastructure will be crucial in making QCDs a viable option in cancer therapy.

## Future directions and emerging trends

The future prospects of CDs are very intriguing from where we stand now. Further developments in this field should make it possible to achieve flexible theranostic purposes in anti-cancer as well as in other diseases therapy due to the multifunctionality of these materials; it’s lesser cost as well as environmental impact, less toxicity, better cell permeability, excellent water solubility, more biocompatibility, high acuity of imaging using florescence probes, and ease of conjugation with treatments. They find applications across diverse fields such as energy storage, chemical sensing, and drug delivery. Synthetic nucleic acid ligands known as aptamers (Apts) are designed to bind to a wide range of substances, such as proteins, amino acids, and medications [188]. They are removed via amplification, recovery, and adsorption techniques from combinatorial libraries of synthetic nucleic acids. Integrating aptamers with nanomaterials enhances their utility in bioanalysis and biomedicine. Nanomaterials associated with Apts, such as liposomes, polymers, dendrimers, carbon-based materials, silica, nanorods, magnetic nanoparticles, and quantum dots (QDs), serve as promising tools in biomedicine [188].

Following conjugation with functional groups and surface changes, these nanomaterials allow for efficient apta-sensing. In advanced biological assays, QDs immobilized with Apts through physical interactions or chemical bonds form the basis of modern QD aptasensing platforms. These platforms depend on the affinity among QDs, Apts, and their target for detection.

QD-Apt association have demonstrated significant potential in directly detecting various cancers including prostate, ovarian, colorectal, and lung cancers, as well as detecting bio-markers involved in these tumorigenesis such as mucin 1, prostate-specific antigen, Tenascin-C, nucleolin, growth factors, prostate-specific membrane antigen, and exosomes. Additionally, Apt-conjugated QDs show promise in combating bacterial infections caused by *Bacillus thuringiensis, Pseudomonas aeruginosa *etc*.* Additionally, xylose, glucose, sucrose, and table sugar have been effectively used to create CDs using a batch hydrothermal technique [165, 188].

Improving the Quantum Yield of carbon-based QDs and adjusting emissions for the application of their intrinsic features is one of the major issues with regard to CQDs and GQDs. It is crucial to produce these QDs based on carbon with strong near-infrared emission for the aim of cell and texture bio-imaging. The majority of these QDs are typically bluish to greenish in colour. Future studies will play a vital role in enhancing these materials by broadening the range of wavelengths covered by GQDs and CQDs to encompass all visible light, particularly in the near-infrared (NIR) spectrum. While the varieties of CQDs are currently more varied than those of semiconductor QDs, but they have a lesser fluorescent QY. Hence, further research is still needed to fully understand their fluorescent/luminescent mechanism. In general, research on carbon-based QDs also focuses on large-scale fabrication of the particles with controlled sizes, quick purification, surface functionalization, and modification. It also continuously explores how to combine these properties with other technologies, such as immunoassays and instrumental analysis. Specifically, carbon nanomaterials with a range of physicochemical characteristics have the potential to induce harmful pharmacological and biological consequences. Thus, one major and difficult problem for medical and biomedical applications is how compatible these nanomaterials are with biosystems. For their clinical application, a number of difficult problems are necessary, such as organ accumulations, cellular toxicity, immunogenicity, uncontrollable agglomeration, incompatibility with biomedia, immunogenicity, and biological persistency in biosystems. Thus, additional a biological model is required for better understanding of biological interactions between carbon nanomaterials and their theranostic benefits. This research should also focus in in vivo monitoring as well as identifying the different underlying properties of these interactions. Considering the observed reduced toxicity compared to instances involving the direct application of raw or pure nanomaterials, future research should prioritize investigating functionalized and surface-modified nanomaterials, such as coating or modifying surfaces with biocompatible and bioactive macromolecules like chitosan, gelatin, PEG, peptides, and others. Furthermore, it is possible to prevent hydrophobic interactions between these carbon-based nanomaterials and cells by functionalizing them either noncovalently or covalently with hydrophilic and biocompatible polymers. Hence it resolving the issues of toxicity and low biocompatibility. Using therapeutically authorised nanotheranostics made of biocompatible, biodegradable, and biorenewable materials like vegetable-oils, bioproducts of animals, etc. is another recommendation. For example, several liposomes, dendrimers, and/or biocompatible polymers have been given clinical approval for usage in different medicinal applications. As a result, it is crucial to conjugate or modify therapeutic (nano)formulations that have already received approval in order to add functional ligands that can enhance or expand their diagnostic index. Clarifying the physicochemical properties of the nanoparticle precursors and nanoparticles themselves at each stage of the synthesis process is essential to developing a safety profile, regardless of the synthesis method used. Precisely characterising the toxicological profiles of these products aids in defining an operational envelope within which these materials may be used safely in the event that such safety parameters cannot be consistently established. Because of this, the establishment of nanosafety requirements, recommendations, and reference points is currently ongoing. Furthermore, because nanoparticles are quantum mechanical in nature, it is not possible to extrapolate reliable standards from physicochemical data collected from macro-sized materials. Since nanoparticles have been hailed as the scientific breakthrough of the future, it stands to reason that fundamental processes and frameworks that apply to conventional materials also apply to nanomaterials, including the tried-and-true method of risk assessment. Finding out the physicochemical properties of nanomaterial precursors and themselves will serve as a first step in risk characterization, enabling a thorough evaluation of any potential hazards that may be present from nanoparticles.

However, despite the immense potential, several challenges must be addressed before quantum-sized carbon dots can be seamlessly integrated into clinical cancer therapies. Concerns regarding biocompatibility, potential toxicity, and long-term effects necessitate thorough investigation. Additionally, optimizing synthesis methods to ensure reproducibility, scalability, and cost-effectiveness is paramount for the translational success of these nanomaterials.

## Conclusion

In conclusion, because of their distinct physicochemical characteristics, carbon nanostructures can incorporate chemotherapeutic drugs, targeting ligands, and a variety of other therapeutics. Literature now firmly establishes the advantages of carbon dots over the majority of nanomaterial types derived from non-carbonaceous sources. Certain superior features, such imaging and therapy, are essential for expanding safety margins in clinical and/or patient-focused applications. Even though CDs are thought to be lesser-toxic, multifunctional NPs, there are a few issues with their utilisation, such as production techniques, managing their size, and surface characteristics. It may interfere with their biological applications. However, due to carbon dots exhibit significant optical absorption, tailored medicine administration and diagnostic imaging are made possible by photo-thermal as well as photo-dynamic treatments, which use the up taken photons to destroy malignant bacteria and tumours selectively. Carbon dots have a plethora of potential industrial uses, including as light-emitting diodes, medical agents, catalytic agents, nucleus-targeted delivery, energy storage media, fluorescence and optic probes, and more. However, each of these uses will require an independent assessment of the toxicity profile of the agents. The results of our investigation indicate that the formulation may be just as significant as the carbon dot itself, if not more so. The selection of recent research covered here suggests and shows that further, more in-depth research is needed, if not absolutely required, to prove beyond a reasonable doubt that carbon dots and other nanomaterials are safe for the environment as well as for the health of people and animals. While QDs give a broad spectrum of usage in cell imaging and research, challenges related to toxicity and pharmacological concerns have impeded progress in cancer therapy and detection. Notably, issues such as substantial colloidal instability and metal insecurity have been identified. Although these issues might not present major challenges for in vitro applications, they severely restrict the use of QDs for in vivo imaging of malignant development.

Developing stronger and biocompatible nanomaterials is crucial for advancing in vivo applications. It is very particular in case of photothermal therapy applications involving both therapy and diagnosis. In the foreseeable future, major advancements are anticipated in this rapidly expanding subject. Moreover, challenges such as the degradation of the coating shell due to changes in quantum dots need to be addressed. Efforts should be directed toward creating distinct QDs with varying compositions, sizes, surface coatings, and valences to mitigate toxic properties and enhance detection. It becomes essential to do a thorough analysis of PL procedures for distinct CD types in order to overcome this problem. The paper offers suggestions for improving surface functionalization of CDs to increase their usefulness. Current literature has unveiled diverse physicochemical characteristics of CDs by proof-of-concept analysis, which hold significance in imaging.

Despite the positive outlook on CD applications, certain aspects remain to be elucidated. The exact process by which cells absorb substances and the potential long-term health effects are still not completely understoodBiodistribution and pharmacokinetics are complex for CDs and are contingent on numerous characteristics like morphology, physicochemical characteristics, formulation, and exterior chemistry. As these field advances, addressing these nuances will be pivotal for unlocking the full potential of CDs in the realm of optical imaging and cancer theranostics.With so many new directions and chances provided by QCDs' special qualities, we see a bright future for these kinds of nanomaterials. Using a range of QCDs, we have presented an extensive overview of the latest developments in biological imaging and nanomedicine therapy in this review study.

## Data Availability

No datasets were generated or analysed during the current study.
